# Evasion of immunosurveillance by genomic alterations of PPARγ/RXRα in bladder cancer

**DOI:** 10.1038/s41467-017-00147-w

**Published:** 2017-07-24

**Authors:** Manav Korpal, Xiaoling Puyang, Zhenhua Jeremy Wu, Roland Seiler, Craig Furman, Htoo Zarni Oo, Michael Seiler, Sean Irwin, Vanitha Subramanian, Jaya Julie Joshi, Chris K. Wang, Victoria Rimkunas, Davide Tortora, Hua Yang, Namita Kumar, Galina Kuznetsov, Mark Matijevic, Jesse Chow, Pavan Kumar, Jian Zou, Jacob Feala, Laura Corson, Ryan Henry, Anand Selvaraj, Allison Davis, Kristjan Bloudoff, James Douglas, Bernhard Kiss, Morgan Roberts, Ladan Fazli, Peter C. Black, Peter Fekkes, Peter G. Smith, Markus Warmuth, Lihua Yu, Ming-Hong Hao, Nicholas Larsen, Mads Daugaard, Ping Zhu

**Affiliations:** 1 H3 Biomedicine Inc., 300 Technology Square, Cambridge, MA 02139 USA; 2 Department of Urologic Sciences, University of British Columbia, Vancouver, BC Canada V5Z 1M9; 3 Vancouver Prostate Centre, Vancouver, BC Canada V6H 3Z6; 4 Eisai Inc., 4 Corporate Drive, Andover, MA 01810 USA; 5 Department of Urology, University Hospital of Southampton, Hampshire, SO16 6YD UK; 6 Department of Urology, University of Bern, Bern, CH-3010 Switzerland

**Keywords:** Cancer genomics, Immunosurveillance

## Abstract

Muscle-invasive bladder cancer (MIBC) is an aggressive disease with limited therapeutic options. Although immunotherapies are approved for MIBC, the majority of patients fail to respond, suggesting existence of complementary immune evasion mechanisms. Here, we report that the PPARγ/RXRα pathway constitutes a tumor-intrinsic mechanism underlying immune evasion in MIBC. Recurrent mutations in RXRα at serine 427 (S427F/Y), through conformational activation of the PPARγ/RXRα heterodimer, and focal amplification/overexpression of PPARγ converge to modulate PPARγ/RXRα-dependent transcription programs. Immune cell-infiltration is controlled by activated PPARγ/RXRα that inhibits expression/secretion of inflammatory cytokines. Clinical data sets and an in vivo tumor model indicate that PPARγ^High^/RXRα^S427F/Y^ impairs CD8^+^ T-cell infiltration and confers partial resistance to immunotherapies. Knockdown of PPARγ or RXRα and pharmacological inhibition of PPARγ significantly increase cytokine expression suggesting therapeutic approaches to reviving immunosurveillance and sensitivity to immunotherapies. Our study reveals a class of tumor cell-intrinsic “immuno-oncogenes” that modulate the immune microenvironment of cancer.

## Abstract (web summary)

Muscle-invasive bladder cancer (MIBC) is a potentially lethal disease. Here the authors characterize diverse genetic alterations in MIBC that convergently lead to constitutive activation of PPARgamma/RXRalpha and result in immunosurveillance escape by inhibiting CD8+ T-cell recruitment.

## Introduction

Cancer is associated with cell autonomous and microenvironmental aberrance, including intrinsic genomic/epigenetic alterations and evasion of immunosurveillance^1,2^. Classic oncogenes with hotspot mutations demonstrate robust cancer cell autonomous transformation and “oncogene addiction” abilities, exemplified by enhancing proliferation/evading apoptosis and exhibiting dependence on the target for growth/survival^3,4^. Therefore, targeted therapy has been widely administered to cancer patients with defined genomic alterations^5^. Interestingly, some of the recently uncovered potential cancer genes with hotspot mutations, e.g., *SPOP* and *U2AF1*, appear to exhibit fewer traits characteristic of the typical oncogenes such as promoting cancer cell growth or conferring oncogene dependence^6,7^. Based on the recurrent nature of the genetic alterations, it is sensible to propose that these non-classical oncogenes may function beyond modulating tumor-intrinsic properties and instead may modulate immune/stromal cell function to promote a favorable microenvironment for tumor growth.

Cancer immunosurveillance constitutes an important host protection mechanism to prohibit cancer progression and recent clinical success of immunotherapies has attracted substantial interest in immune-directed anti-cancer strategies^8–10^. Although immune checkpoint blockade, e.g., anti-CTLA4, anti-PD-1 or anti-PD-L1 treatment, has shown promising results in the clinic, usually only a fraction of patients respond to the treatment^11,12^. Understanding the mechanisms underlying intrinsic or acquired resistance to immunotherapy is pivotal for expanding the clinical benefits to more cancer patients. It has been suggested that total mutation burden, potential neoantigen load, and status of immune cell infiltrates and cytokine levels may have influence on the response of cancer patients to immunotherapy^13–17^. In addition, emerging evidence suggests that metabolic and cancer cell intrinsic oncogenic signals may mediate cancer immune evasion and resistance to immunotherapies^18–22^. Hence, in addition to understanding the regulatory roles of immune cells in cancer, elucidation of the intrinsic genomic alterations in cancer cells that may impact immune status or reprogramming of the cancer microenvironment is key to deciphering molecular mechanisms that limit response to immunotherapies in the clinic.

Here, we report that hotspot S427F/Y mutations in *RXRA* or focal amplification/overexpression of *PPARG* in bladder cancer induce the activation of the PPARγ/RXRα pathway, leading to suppression of cytokine secretion from cancer cells. Non-inflamed immune phenotypes are observed in human bladder tumors and in a syngeneic mouse bladder tumor model. Likely as a consequence of the suppressive immuno-environment, we further demonstrate that PPARγ/RXRα pathway activity in the syngeneic bladder tumor model renders partial resistance to immune checkpoint blockade suggesting the potential for PPARγ/RXRα signaling to promote innate/acquired resistance to immunotherapies in the clinic. Collectively, we posit that this resistance phenotype may be reversed by therapeutic targeting of PPARγ/RXRα offering a therapeutic approach to sensitizing bladder cancer with activated PPARγ/RXRα pathway to immunotherapies.

## Results

### Genomic alterations of RXRA and PPARG inbladder cancer

**Fig. 1:**
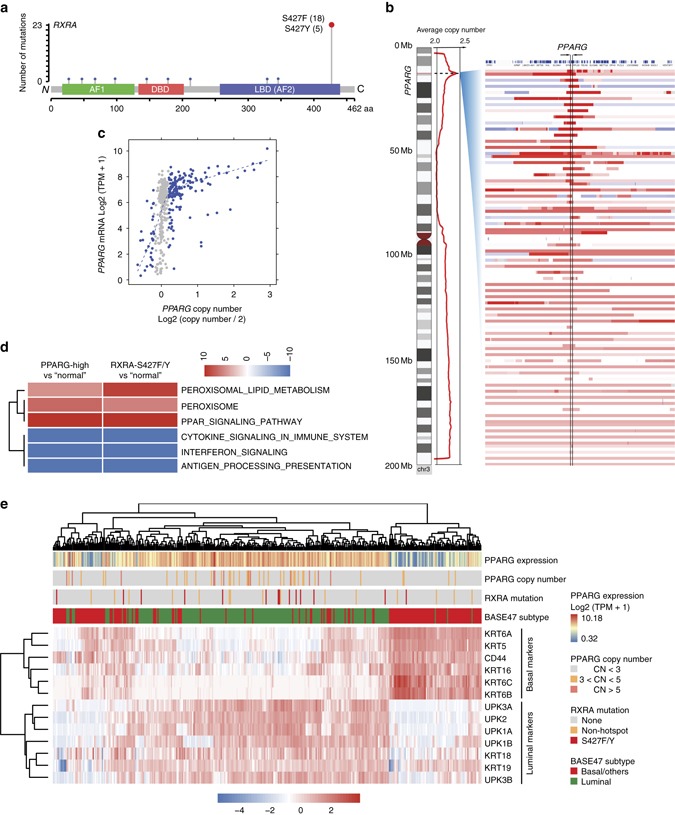
Recurrent genomic alterations of *RXRA* and *PPARG* in bladder cancer. **a** Hotspot S427F/Y mutations of *RXRA* in TCGA muscle-invasive bladder cancer (*MIBC*) cohort, BGI/Shenzhen bladder cancer cohort and DFCI/MSKCC bladder cancer cohort (*n* = 534). **b** Focal amplification of *PPARG* in TCGA MIBC. **c** Correlation of copy number (*CN*) and mRNA expression of *PPARG* in TCGA MIBC (*P* < 2.2e−16). *Gray dots* represent those samples without CN changes (2.30>CN>1.74). **d** Pathway enrichment analysis of *RXRA-S427F/Y* and *PPARG-high* in bladder cancer relative to “normal” *RXRA-WT* and non-hotspot or *PPARG-low*. **e** Distribution of *PPARG* mRNA expression, *PPARG* copy number variation (*CNV*), and *RXRA* mutations in subtypes (BASE47 luminal/basal) of TCGA bladder cancer (*n* = 385). Statistical analysis was performed using Fisher’s exact test and *P* < 0.05 was considered as statistically significant

Performing comprehensive mining of cancer genomic alterations in The Cancer Genome Atlas (TCGA) and other cancer genomic databases, we observed RXRA^S427F/Y^, located in the ligand-binding domain (LBD) of RXRα, as hotspot mutations specifically enriched in bladder cancer (4.3%, 23/534)^23–25^ (Fig. 1a, Supplementary Fig. 1). Since RXRα forms heterodimers with class II nuclear receptors (NRs) to regulate their transcriptional activities^26,27^, we analyzed differentially expressed gene programs in RXRα^S427F/Y^ compared with wild-type (WT) tumors as well as genomic status and mRNA expression of all class II NRs that are known to heterodimerize with RXRα^27^ using TCGA data set. The most significant differentiation between WT and S427F/Y mutant tumors included upregulation of peroxisome proliferator-activated receptors (PPARs)-regulated gene expression involved in lipid and peroxisome metabolism in the mutant setting (Supplementary Fig. 2). Moreover, *PPARG*, the gene encoding PPARγ, is the only RXRα-heterodimerizing NR gene that exhibited recurrent genomic alterations (amplification) and high expression selectively in muscle-invasive bladder cancer (MIBC) among the cancer types profiled by TCGA (Supplementary Fig. 3). Located at the center of the focal amplicon in chromosome 3p (Fig. 1b), *PPARG* was identified as a significant focal amplification specifically in bladder cancer by genome identification of significant targets in cancer (GISTIC) analysis^28^. The amplification of *PPARG* (copy number (CN)>4) occurred in 12% (49/410) of the TCGA MIBCs. Importantly, amplification of *PPARG* was associated with increased mRNA expression of *PPARG* (*P* < 2.2e−16), suggesting that it is functionally critical (Fig. 1c). Interestingly, overexpression of *PPARG* (log TPM > 7) also occurred in about 27% (96/358) MIBC patients without CN gain (CN<3), suggesting that a subset of the bladder tumors may engage non-genomic mechanisms to facilitate high expression of *PPARG*. In aggregate, nearly 40% of MIBC show genetic lesions or non-genomic based misexpression in the RXRα/PPARγ heterodimer suggesting an essential role for this complex in the pathogenesis of MIBC.

We next analyzed the transcriptional impact of these genetic alterations by stratification into three groups in the TCGA MIBC cohort. We found a significant overlap of the differentially expressed genes in the *RXRA*-S427F/Y and *PPARG*-overexpressed (Log_2_(TPM + 1)>7) groups relative to the “normal” group (*RXRA*-WT or non-S427F/Y, *PPARG* Log_2_(TPM + 1)≤7), with lipid metabolism and PPAR pathways being commonly activated in both *RXRA* and *PPARG* alterations (Fig. 1d, Supplementary Fig. 4). Interestingly, cancer immunity-related cytokine and antigen processing/presentation pathways were commonly downregulated in MIBC bearing *RXRA* or *PPARG* alterations (Fig. 1d, Supplementary Fig. 4). These data suggest that both genomic alterations act on convergent pathways to potentially drive development of bladder cancer. We also explored the possibility that *RXRA* or *PPARG* alterations may be restricted to subtypes of bladder cancer classified by expression and genomic profiles^23,29–32^. Indeed, *RXRA*^S427F/Y^ (*P* = 0.037), *PPARG*-amplification (CN > 3, *P* = 5.426e−05), and overexpression (Log_2_(TPM + 1)>7, *P* < 2.2e−16) were significantly enriched in the luminal subtype of MIBC as assessed by a Fisher’s exact test, while *RXRA*^S427F/Y^ and *PPARG* amplification/overexpression trended toward mutual exclusion (Fig. 1e). Taken together, this suggests that the non-overlapping recurrent genomic alterations of *RXRA* and *PPARG* may similarly play a crucial role in a subset of luminal MIBC.

### RXRα^S427F^ enhances interaction with and activation of PPARγ

**Fig. 2:**
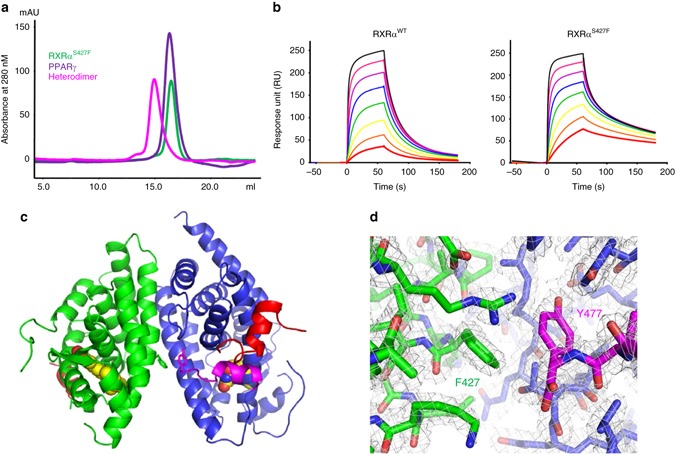
S427F mutation in RXRα stabilizes heterodimerization with PPARγ and promotes the agonistic conformation. **a** Sizing profile of RXRα ^S427F^ mutant (*green*), PPARγ (*purple*), and the heterodimer (*magenta*). Both RXRα^S427F^ and PPARγ run as monomers. When mixed together in 1:1 stoichiometry, the elution profile shifts demonstrating formation of the heterodimer in the absence of ligand. **b** SPR demonstrating enhanced interaction between RXRα ^S427F^ mutant and PPARγ. RXRα was immobilized to the CM5 chip by amine coupling and PPARγ was injected in dose response from 3 μM to 24 nM with 60 s association phase and 120 s disassociation. **c** Overall crystal structure of the heterodimer complex of RXRα^S427F^ mutant (*green*) and PPARγ (*blue*) with the co-activator peptide Src1 (*red*). The agonists 9-cis-retinoic acid and rosiglitazone are rendered as spheres. The AF-2 helix (Helix H12) of PPARγ has been highlighted in *magenta*. RXRα^S427^ and PPARγ^Y477^ are rendered as sticks and located in the dimer interface. **d** Zoom in of the heterodimer interface shows the S427F mutation of RXRα (*green*) introduces a π-stacking interaction with Y477 of PPARg (*blue*) at the C-terminus (*magenta*). The 2Fo–Fc electron density map is shown in *gray* and contoured at 1.2 s

To determine the functional impact of RXRα^S427F/Y^ we next aimed to gain structural insights into how the mutations may alter its ligand binding or heterodimerization properties with PPARγ. Available crystal structures show that the S427 residue is located in the dimer interface, outside the ligand binding pocket (Supplementary Fig. 5), suggesting that the mutations are unlikely to alter the ligand binding properties of RXRα. Instead, the mutations could alter the selectivity profile for other NR partners and/or the oligomerization properties. We analyzed the available heterodimer crystal structures to predict, in silico, how the mutation might impact the selectivity profile for other NR partners (Supplementary Fig. 6). We predicted that the mutation would be accommodated, having little impact, in RXRα heterodimers with many partner receptors, such as LXR, PXR, RAR, CAR, and TR. The mutation might be detrimental to heterodimers with FXR and VDR, and homodimer of RXRα itself (Supplementary Figs. 5b, and 6), due to steric interference. However, we predicted that RXRα heterodimer with PPARγ would be enhanced in the mutant owing to accommodation of S427F/Y at the dimer interface and a likely newly formed π–π stacking between the mutant residue and Y477 of PPARγ in the C-terminus (Supplementary Fig. 6b). Since the S427F is the prevalent mutation found in patients (Fig. 1a), we focused on this for additional characterization by purifying the LBD of RXRα^WT^ and RXRα^S427F^ (Supplementary Fig. 7a). Consistent with previous studies^33,34^, we confirmed that RXRα^WT^ eluted predominantly as a tetramer and dissociates to monomer with addition of 9-cis-retinoic acid (Supplementary Fig. 7b). Interestingly, RXRα^S427F^ elutes exclusively as a monomer in the absence of any exogenous ligand (Fig. 2a, Supplementary Fig. 7c). Examination of the dimer interface showed that S427 was tucked into a small hydrophilic pocket and substitution with the bulky phenylalanine or tyrosine would disrupt this dimer (Supplementary Figs. 5 and 6). Moreover, we found that the mutant was able to associate with PPARγ in a ligand-independent fashion (Fig. 2a). We next characterized the interaction between PPARγ and monomeric RXRα^WT^ or RXRα^S427F^ using surface plasmon resonance (SPR). RXRα^S427F^ showed enhanced binding affinity to PPARγ than its WT counterpart (Fig. 2b, Supplementary Fig. 8). Together, these data suggest the S427F primes RXRα by altering its oligomerization state and enhancing its interaction with PPARγ.

To further characterize this mutation, we determined the crystal structure of the heterodimer RXRα^S427F^ and PPARγ bound to 9-cis-retinoic acid and rosiglitazone, respectively, with co-activator peptide Src1 at 1.95 Å resolution (Fig. 2c, Supplementary Table 1). Overall, our structure superimposed closely on the WT structure^35^ (PDB 1FM6) with a RMS of 1.4 Å for all Cα π–π stacking interaction with the C-terminal Y477 of PPARγ resulting in 11 new Van der Waals (VDW) contacts (Fig. 2d). These new interactions may account, in part, for the tighter binding and slower off-rate observed in the SPR experiments. The mutation did not appear to significantly alter the ligand binding pockets, but the additional interactions with Y477 could have a stabilizing effect on the active conformation of AF-2 (Fig. 2d). In aggregate, our data indicate that the S427F mutation primes RXRα in the monomeric form and enhances its interaction with PPARγ, potentially predisposing PPARγ for binding co-activator partners. These aberrant properties of RXRα^S427F^/PPARγ could contribute to both activation of PPARγ-dependent transcription and transrepression of inflammation/immune-related genes^36^.

### RXRα^S427F/Y^ or PPARγ overexpression activates PPARγ pathway

**Fig. 3:**
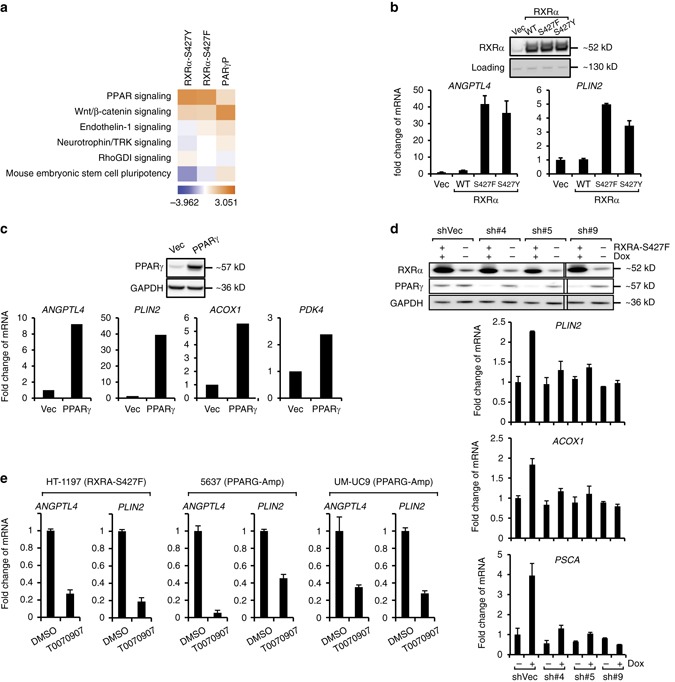
RXRα^S427F/Y^ functionally promotes ligand-independent PPARγ signaling in human bladder cancer lines. **a** Heat map representing pathways activated/suppressed in RXRα^S427Y^, RXRα^S427F^ and PPARγ overexpressing lines relative to their respective controls. *Orange* represents pathway activation and *blue* represents pathway suppression. The analysis was based on three biological replicates. **b** *Upper*, western blot of RXRα confirming overexpression of RXRα^WT^ (WT), RXRα^S427F^ (S427F) and RXRα^S427Y^ (S427Y) in T24 cells relative to control (Vec). *Lower*, RT-qPCR analysis of *ANGPTL4* and *PLIN2* in various engineered lines. **c** *Upper*, western blot confirming overexpression of PPARγ in T24 line relative to control (Vec). *Lower*, RT-qPCR analysis of *ANGPTL4*, *PLIN2*, *ACOX1* and *PDK4* in engineered lines. **d** *Upper*, western blot of RXRα and PPARγ in SV-HUC line engineered to inducibly overexpress RXRα^S427F^ and knockdown PPARγ by multiple shRNAs (sh#4, 5 and 9) upon doxycycline (*DOX*) treatment. *Lower*, RT-qPCR analysis of *PLIN2*, *ACOX1* and *PSCA* in various SV-HUC-1 engineered lines. +/− represents presence or absence of DOX treatment respectively. **e** RT-qPCR analysis of *ANGPTL4* and *PLIN2* in HT-1197 (carrying endogenous RXRA^S427F^), 5637 and UM-UC9 (PPARG amplified) lines treated with DMSO or T0070907 for 24 h. All RT-qPCR data is normalized to *GAPDH* and presented as mean fold change vs. control ± SEM of at least three biological replicates

We next examined the potential for RXRα^S427F/Y^ to promote PPARγ function in the cellular setting. Global transcriptome profiling revealed a significant overlap of differentially expressed genes between the PPARγ, RXRα^S427F^ and RXRα^S427Y^ overexpressing human bladder cancer lines relative to their respective controls (Fig. 3a, Supplementary Fig. 9). Furthermore, as we observed in the TCGA MIBC data set (Supplementary Fig. 4), all three genetic alterations commonly regulated a number of pathways with PPAR signaling being the most significantly activated (Fig. 3a). Supporting this finding, overexpression of RXRα^S427F/Y^ but not RXRα^WT^ significantly enhanced ligand-independent expression of known PPARγ target genes *ANGPTL4* and *PLIN2*^37,38^ in several human bladder cancer lines (Fig. 3b, Supplementary Fig. 10a) and in the immortalized normal bladder line SV-HUC-1 (Supplementary Fig. 10b), confirming the general role of RXRα^S427F/Y^ in regulating PPARγ function. We confirmed *ANGPTL4* and *PLIN2* as bona fide PPARγ target genes in bladder lines as pharmacological modulation by PPARγ agonist rosiglitazone (Rosi) or antagonist T0070907 significantly altered the expression of these genes (Supplementary Fig. 10b). Furthermore, PPARγ overexpression (Fig. 3c, Supplementary Fig. 11) also enhanced the ligand-independent expression of PPARγ target genes^39,40^ suggesting that all three genetic alterations converge onto the PPARγ pathway.

To assess whether RXRα^MUT^ are dependent on PPARγ for transcriptional regulation, we engineered SV-HUC-1 lines to inducibly express RXRα^S427F^ and shRNAs targeting PPARγ. Whereas the control shRNA (shVec) showed a significant increase in expression of target genes following expression of RXRα^S427F^, the increase in expression was significantly blunted following knockdown of PPARγ (Fig. 3d). We further confirmed this dependence using PPARγ-specific antagonist T0070907 in human bladder cancer lines HT-1197 carrying the endogenous RXRα^S427F^, and 5637 and UM-UC9^41^ lines bearing PPARγ amplifications (Fig. 3e, Supplementary Fig. 12). Therefore, RXRα^MUT^ preferentially and constitutively interact with PPARγ to activate PPARγ/RXRα-mediated signaling activity in the bladder cancer cells.

Having confirmed that RXRα^MUT^ and PPARγ alterations both constitutively activate PPARγ alterations dysregulation α^S427F/Y^ or PPARγ overexpression (Supplementary Fig. 13a). Furthermore, an insignificant growth inhibition was observed following PPARγ knockdown across a number of cell lines (Supplementary Fig. 13b, c). This insignificant effect on growth following genetic knockdown of PPARγ was further confirmed by pharmacologically inhibiting the pathway across a variety of cell lines spanning multiple genotypes, suggesting this pathway may have insignificant influence on growth-promoting cancer phenotypes in vitro (Supplementary Fig. 14).

### PPARγ/RXRα^S427F/Y^ is associated with non-inflamed phenotype

**Fig. 4:**
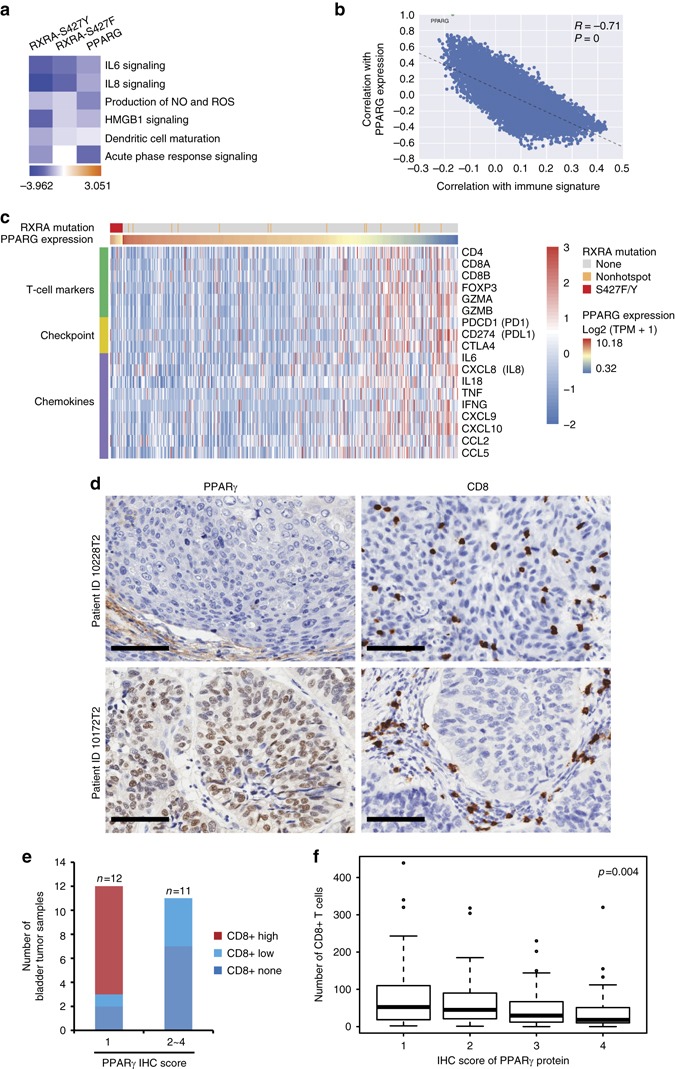
Tumor-intrinsic activation of PPARγ/RXRα is negatively correlated with immune infiltration. **a** Pathway enrichment analysis of genes differentially expressed in RXRA-S427Y, RXRA-S427F and PPARG overexpressing T24 lines relative to respective controls. Top suppressed pathways are shown. The analysis was based on three biological replicates. **b** Dot plot showing expression correlation of all genes with the curated immune signature (refer to “Methods”) vs. correlation with PPARG in bladder tumors (*n* = 385) from TCGA. **c** Heatmap presenting associations between RXRA mutations and PPARG expression with T-cell markers (*top, green label*), immune checkpoint molecules (*middle, yellow label*), and pro-inflammatory factors (*bottom, lavender label*) in TCGA MIBC (*n* = 385). **d** IHC staining of PPARγ and CD8 in two representative human bladder tumor samples from a clinical cohort (*n* = 23, Eisai cohort). Scale bars: 100 μm. **e** Summary of the IHC results of Eisai cohort shown in **d**. Distribution of CD8+ T-cell infiltration in bladder tumors expressing high (scores 2–4) or low (score 1) levels of PPARγ protein. **f** Whisker plot representing IHC staining of infiltrating CD8+ T cells and PPARγ protein expression of MIBC samples from the bladder cancer meta-dataset (*n* = 118). No expression, score = 1; High expression, score = 4. The bold lines: median; the boxes: interquartile range (*IQR*); the upper whiskers: min(max(x), Q_3 + 1.5 * IQR); the lower whiskers: max(min(x), Q_1−1.5 * IQR). Statistical analysis was performed using Kruskal–Wallis test

As noted previously, RXRα ^S427F/Y^ and PPARγ-overexpressing bladder tumors were significantly associated with suppression of immune infiltration and inflammation-related pathways (Fig. 1d). In support of this, we found that cytokine and immune pathways were significantly suppressed by PPARγ or RXRα^S427F/Y^ in the engineered T24 bladder cancer cell lines indicating that tumor autonomous signaling may influence the host immune response (Fig. 4a). We further performed a correlation analysis across the TCGA bladder tumors for PPARγ and a curated immune signature including inflammatory factors and immune lineage markers (refer to “Methods”). Overwhelmingly, PPARγ-associated expression was significantly anti-correlated with the immune signature hinting at the possibility that tumor-autonomous PPARγ activity may suppress cytokine secretion and immune cell infiltration (Fig. 4b). In addition to high PPARγ expressing tumors, RXRα^S427F/Y^ positive bladder tumors also showed an anti-correlation with immune lineage markers, immune checkpoint genes, and inflammatory chemokines suggesting that both genotypes may significantly hamper immune infiltration (Fig. 4c, Supplementary Fig. 15).

To further validate this finding, we extended our analysis to additional clinical data sets. To this end, we identified PPARγ active and inactive tumors using GLMnet74 (refer to “Methods”) across an independent cohort of 127 chemotherapy naïve MIBC (TURBT meta-dataset) and the MD Anderson bladder cancer data set (GSE48075). Following identification of PPARγ active/inactive tumors, we applied gene set enrichment analysis (GSEA) using hallmark gene sets (MSigDB Collections) and a bladder cancer-specific immunesignature190 (refer to “Methods”). In support of the TCGA-centric analysis above (Fig. 4 b,c, Supplementary Fig. 15), this analysis also revealed that immune/inflammation-related hallmark gene sets and immunesignature190 were negatively enriched in PPARγ active bladder cancers in two independent cohorts (Supplementary Figs. 16 and 17). Additionally, *PPARG* expression in bladder cancer was significantly correlated with reduced expression of T-cell markers, immune checkpoint genes and inflammatory factors (Supplementary Fig. 18), indicating that tumor-intrinsic PPARγ activity might suppress the tumor immune surveillance by excluding immune cell infiltration in bladder cancer.

We performed immunohistochemistry (IHC) staining for PPARγ and CD8 in a cohort of human bladder tumors (Eisai cohort) (Supplementary Table 2). This analysis revealed a significant anti-correlation between PPARγ expression in tumor cells and infiltration of CD8+ T cells into the tumor compartment (Fig. 4 d,e, Supplementary Fig. 19, Supplementary Table 3). We further performed similar PPARγ and CD8 staining in an independent larger cohort of bladder tumor samples (bladder cancer meta-dataset). Again, we observed a significant decrease in infiltrating CD8+ T cells into PPARγ^High^ tumors (*P* = 0.004, Kruskal–Wallis test) (Fig. 4f, Supplementary Fig. 20). In summary, gene expression and protein staining across several independent clinical cohorts demonstrate that PPARγ^High^/RXRγ^S427F/Y^ tumors are associated with less CD8+ T-cell infiltration and non-inflamed immune microenvironment.

### PPARγ/RXRα^S427F/Y^ blunts immunotherapy response

**Fig. 5:**
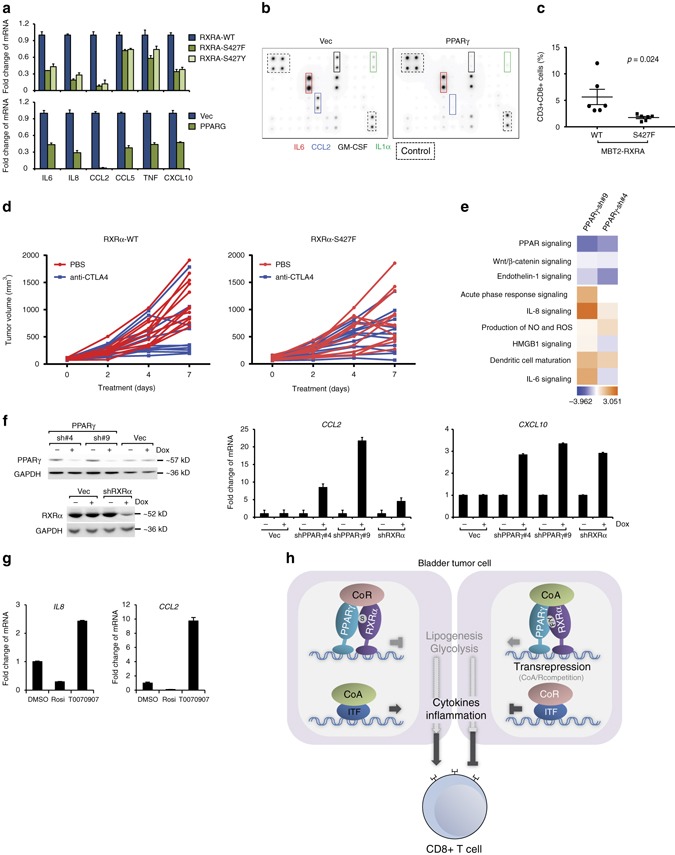
PPARγ/RXRα^S427F^ confers partial resistance to immunotherapies. **a** RT-qPCR analysis of chemokines/cytokines in T24 lines engineered to overexpress RXRA-WT, RXRA-S427F, RXRA-S427Y (*upper*), and PPARG (*lower*). Controls are RXRA-WT for RXRA mutant lines and vector control (Vec) for PPARG overexpressing line. Expression normalized to *GAPDH* and data presented as mean fold change vs. control ± SEM of three biological replicates. **b** Chemokine array analysis of conditioned media collected from T24 lines engineered to overexpress PPARG (PPARγ) vs. control (Vec). Dotted boxes represent controls. Significant changes in secretion are outlined. One representative of three independent experiments is shown. **c** FACS based quantitation of infiltrating CD3 + CD8 + double positive T cells into subcutaneously implanted MBT2 tumors overexpressing RXRA-WT (*n* = 6) or RXRA-S427F (*n* = 6). Data presented as percent of total tumor-derived cells following dissociation. **d** *Left*, individual MBT2-RXRα^WT^ tumor volumes in response to PBS (*red*, *n* = 12) or anti-CTLA4 (*blue*, *n* = 12). *P* = 0.0189 at day 7. *Right*, individual MBT2-RXRα^S427F^ tumor volumes in response to PBS (*red*, *n* = 12) or anti-CTLA4 (*blue*, *n* = 12). *P* > 0.05 at day 7. One-way ANOVA followed by Tukey’s post-hoc test performed. **e** Heatmap presenting pathway level analysis (activation, *red*; suppression, *blue*) of differentially expressed genes in PPARγ knockdown lines (PPARγ-sh#4 and -sh#9 engineered in SV-HUC-1 line expressing RXRA-S427Y) relative to vector control. The analysis was based on three biological replicates. **f** *Left*, Knockdown of PPARγ or RXRα by shRNAs in HT-1197 cells. GAPDH was used as the control. *Right*, RT-qPCR analysis of inflammatory genes *CCL2* and *CXCL10* following inducible knockdown of PPARγ and RXRα in HT-1197 cells. Data normalized to *GAPDH* and presented as mean fold change (Dox treated vs. untreated) ± SEM of three biological replicates. **g** RT-qPCR analysis of *IL8* and *CCL2* following treatment with PPARγ agonist rosiglitazone (Rosi) or PPARγ antagonist T0070907 in 5637 cells. Data normalized to *GAPDH* and presented as mean fold change ± SEM of three biological replicates. **h** Schematic representation of the role of tumor-intrinsic PPARγ/RXRα^S427F/Y^ in transcriptional regulation and immunosurveillance. CoA, co-activator complex; CoR, co-repressor complex; ITF, inflammation-related transcription factors

We probed the potential mechanism(s) that promote the non-inflamed immuno-phenotype by PPARγ/RXRα^S427F/Y^ in bladder cancer. Previous analysis revealed the potential for PPARγ/RXRα^S427F/Y^ to influence inflammatory gene expression (Fig. 4a, Supplementary Fig. 18). To functionally confirm the potential for PPARγ/RXRα^S427F/Y^ to influence chemokine expression and secretion in bladder tumor cells, we profiled the expression/secretion of key inflammatory factors in engineered T24 lines *in vitro*. Consistent with the correlation analysis, RXRα^S427F/Y^ or PPARγ overexpression significantly inhibited expression (Fig. 5a) and secretion (Fig. 5b, Supplementary Fig. 21) of key pro-inflammatory chemokines that function as chemoattractant of effector T cells, including IL6, IL8, CCL2, CCL5, TNF, and CXCL10. CXCL10 has been associated with better reponse to anti-PDL1 treatment of bladder cancer patients in clinical trials^16^. Collectively, our findings suggest that the PPARγ/RXRα^S427F/Y^ pathway in tumor cells might influence the activity/localization of host immune cells and potentially blunt response to immunotherapy. Because many of these genes are transcriptional targets of NFκB, we evaluated the potential regulation of NFκB factors by PPARγ. The analysis shows that the protein level and subcellular localization of the NFκB subunit p65 remain unaffected by PPARγ over-expression (Supplementary Fig. 22). Nevertheless, PPARγ/RXRα could transrepress NFκB target genes by interaction with the promoter of these genes or competing with the transcriptional coregulators as proposed in the literature^36^.

To functionally test the possibility that tumor-intrinsic PPARγ/RXRα^S427F/Y^ pathway activity may influence localization of CD8+ T cells, we subcutaneously implanted murine bladder tumor MBT2 cells engineered to overexpress RXRα^WT^ or RXRα^S427F^ (Supplementary Fig. 23) into syngeneic mice and assessed basal tumor infiltration of CD8+ T cells once the tumors reached ~300 mm^3^. MBT2-RXRα^S427F^ tumors presented less CD3+ CD8+ T-cell infiltration with a greater than twofold decrease relative to WT tumors (Fig. 5c). In contrast, other immunocytes, including T helper (CD3+ CD4+), Treg (CD4+FOXP3+) and monocyte/MDSC (CD11b+), did not show significant alterations of infiltration in RXRα^S427F^ tumors relative to WT tumors (Supplementary Fig. 24). In light of recent reports speculating the importance of CD8+ T-cell infiltration into tumors for driving checkpoint blockade efficacy and the lack of responders to atezolizumab (anti-PD-L1) in the TCGA Cluster I luminal subtype^16^, we also evaluated PPARγ/RXRα activity (GLMnet74, refer to “Methods”) in MIBC from three cohorts (TGCA, bladder cancer meta-dataset and MD Anderson). The vast majority of PPARγ/RXRα active tumors were presented in Cluster I, a subset of luminal bladder cancer, suggesting PPARγ/RXRα activation may contribute to lack of response to immunotherapy (Supplementary Fig. 25). We next assessed functional response of MBT2-RXRα^WT^ and -RXRα^S427F^ tumors to immune checkpoint therapy in the C3H syngeneic mouse model. The MBT2-RXRα^WT^ tumors showed a statistically significant response to anti-CTLA4 treatment at day 7 (*P* = 0.0189, one-way analysis of variance (ANOVA) followed by the Tukey post-hoc test) (Fig. 5d). Similarly, MBT2-RXRα^WT^ also showed a significant response to anti-PD-1 treatment at day 5 (*P* < 0.01, one-way ANOVA followed by the Tukey post-hoc test) (Supplementary Fig. 26). In contrast, MBT2-RXRα^S427F^ tumors showed a partial resistance to both immunotherapies (*P* > 0.05, one-way ANOVA followed by the Tukey’s post-hoc test) (Fig. 5d, Supplementary Fig. 26). Collectively, these data reinforce the anti-inflammatory role of tumor-intrinsic PPARγ/RXRα activity and potentially as a driver of resistance to immune checkpoint inhibitor therapies in bladder cancer.

Having uncovered genomic activation of PPARγ/RXRα pathway as a potential resistance mechanism to immunosurveillance and immunotherapies, we next attempted to explore the impact of therapeutic targeting of RXRα or PPARγ on inflammatory gene expression as an initial step to restore the inflamed immuno-environment and immune-response in bladder cancer. We found that knockdown of PPARγ significantly augmented chemokine-related immune pathway activity in bladder cancer cells (Fig. 5e). Furthermore, chemokines CCL2 and CXCL10, which contribute to immunosurveillance, were induced by shRNAs of PPARγ and RXRα in HT-1197 bladder cancer cells bearing endogenous RXRα^S427F^ (Fig. 5f). In addition, pharmacological inhibition of PPARγ with antagonist T0070907 significantly increased the expression of key cancer immunity-enhancing inflammatory factors including CCL2 and IL8 in the *PPARG*-amplified 5637 cells (Fig. 5g). Together, our data propose that the PPARγ/RXRα^S427F^ pathway may serve as a viable therapeutic node for activating immunosurveillance, enhancing response to immunotherapies in bladder cancer.

## Discussion

In this study we show that S427F/Y hotspot mutations of RXRα and focal amplification or overexpression of PPARγ, occurring in nearly 40% of MIBC, activate PPARγ/RXRα heterodimer function in a ligand-independent manner. We further confirm, through interrogation of multiple engineered lines and clinical data sets, that tumor-intrinsic PPARγ/RXRα pathway activity inhibits host immune response through suppressing expression and secretion of inflammatory factors. Furthermore, we demonstrated the functional role of RXRα^S427F^ in hindering tumor infiltration of CD8+ T cells and promoting partial resistance to checkpoint blockade therapies in a syngeneic tumor model. Lastly, we present evidence that therapeutic targeting of PPARγ may reactivate the immune response and potentially also the response to immunotherapies by enhancing expression of pro-inflammatory factors (Fig. 5h).

We demonstrate that recurrent alterations in PPARγ/RXRα heterodimer lead to enhanced PPARγ signaling, reinforcing the importance of this pathway in bladder cancer biology. Surprisingly, unlike classic oncogenic pathways that promote cancer cell proliferation, PPARγ/RXRα alterations slightly reduced cell growth in vitro (Supplementary Fig. 13). Although we cannot completely exclude the potential role of PPARγ/RXRα in transformed cell autonomous phenotypes, our data suggest that induction of microenvironmental reprogramming favored by tumorigenesis is a key function of PPARγ/RXRα activation in bladder cancer. Thus, RXRA^S427F/Y^ and PPARG-Amp represent an expanding list of tumor cell-intrinsic “immuno-oncogenes” that promote tumorigenesis through modulating the immune microenvironment. Future studies will help to refine the level of tumor-stroma interaction driving tumorigenesis that may ultimately reposition immunotherapies from targeting immune lineages to cancer cells themselves.

Bacillus Calmette–Guérin (BCG) was the first FDA-approved intravesical immunotherapy to treat non-muscle invasive bladder cancer patients by stimulating innate immune responses targeting tumors as bystander to the bacterial threat. Interestingly, PPARγ was suggested to be regulated by and associated with response to BCG^42–44^. Recently, bioinformatics studies implied an anti-correlation between PPARγ expression and T-cell-inflamed phenotypes^45,46^. Clinical trials using immune checkpoint blockade in metastatic bladder cancer have revealed highly encouraging efficacy^47,48^, yet only about 25% of the patients responded to checkpoint inhibitors, such as anti-PD-L1 atezolizumab^16^. Response-predictive biomarker analysis from these studies revealed that high CD8+ T-cell infiltration, mutation burden, and molecular subtypes were associated with better clinical response of bladder cancer patients^16^. Although PPARγ/RXRα status was not correlated with mutation burden in bladder cancer (Supplementary Fig. 15c), there is a significant anti-correlation with CD8+ T-cell infiltration across four independent cohorts of clinical samples (Fig. 4, Supplementary Fig. 18), suggesting that PPARγ/RXRα could serve as an independent marker of immuno-phenotype and responsiveness to immunotherapy. Indeed, using a syngeneic mouse bladder tumor model, we showed that RXRα^S427F^ reduced tumor infiltration of CD8+ T cells, and conferred partial resistance to checkpoint blockade treatment (Fig. 5 c,d, Supplementary Fig. 26).

It has previously been shown that PPARγ/RXRα suppresses immune cell activity by transrepression of pro-inflammatory transcriptional factors^36,49^. Bladder cancer cells appear to utilize a similar molecular strategy to evade immunosurveillance. In bladder cell lines, RXRα^S427F/Y^ or PPARγ overexpression is both sufficient and necessary to downregulate the expression of a series of pro-inflammatory chemokines. Reduced secretion of chemokines may allow the tumors to reprogram the immune microenvironment to be less “inflamed”, consequently enabling resistance to immune-directed therapies (Fig. 5h). Compromising PPARγ/RXRα function through RNAi and pharmacological inhibition enhanced expression of immune-potentiating chemokines in bladder cancer cells bearing RXRα^S427F/Y^ or overexpressing PPARγ, offering the initial steps toward reprogramming the cancer immunity and restoring response to immunotherapies (Fig. 5e–g).

It is well documented that hormones play important physiological roles in homeostasis of metabolism and immunity. Steroids have been widely used as anti-inflammatory treatment in the clinic. PPARγ is a key regulator of lipid and glucose metabolism in many cell types, and also possesses a robust anti-inflammatory activity in immune cells^49,50^. Notably, recent studies on cancer immunity revealed the importance of lipid and glucose metabolism in regulating immune cell activity and cancer immunosurveillance^7,19,51^. Cancer cells can suppress the functionality of T effector cells by competing for glucose sources in the microenvironment^21,22^. In addition, altered triglyceride biosynthesis in dendritic cells or cholesterol metabolism in T cells appears to be a key mechanism to inhibit tumor immunity by inducing T-cell exhaustion or dendritic cell homeostasis^51–53^. Of note, the PPARγ/RXRα pathway has a well-established function in lipid and glucose metabolism in adipocytes and muscle cells^50,54^. Agonistic PPARγ augments glucose uptake and oxidation of fatty acids, two metabolic events recently associated with cancer cell-immune cell energy competition^22,55^. On the other hand, it has been shown that lipid and glucose metabolism also influences cytokine secretion and modulation of inflammation^56–58^. Therefore, it is conceivable that the classic physiological axis of hormone/metabolism/immunity may play a central role in immunomodulation of cancer not only in cell autonomous manners but also through cancer/immune/stromal cell-cell interactions (Fig. 5h). Further understanding of the regulatory mechanisms and deeper identification of relevant pathways/targets in specific tumors should pave the way for the next-generation cancer immunotherapies.

## Methods

### Cell culture

All human cell lines except UM-UC9 were purchased from ATCC. The UM-UC9 cell line was purchased from ECACC. Mouse bladder tumor cell line MBT2 was purchased from the Japanese Collection of Research Bioresources (JCRB) Cell Bank. Cell line authentication was achieved by genetic profiling using polymorphic short tandem repeat (STR) loci (ATCC). All cell lines were free of mycoplasma contamination. Parental and derivative 5637 (PPARγ amplified human bladder cancer line) and KU-19-19 (human bladder cancer line) lines were maintained in ATCC-formulated RPMI-1640 medium containing 10% fetal bovine serum (FBS). Parental and derivative HT-1197 (RXRα^S427F^-bearing human bladder cancer cell line) and SCaBER (human bladder cancer cell line) lines were maintained in ATCC-formulated Eagle’s Minimum Essential medium containing 10% FBS. Human bladder cancer T24 parental and derivative lines and the colorectal cell line HCT-116 were maintained in ATCC-formulated McCoy’s 5a Medium Modified containing 10% FBS. Normal, immortalized SV-HUC-1 cells were maintained in ATCC-formulated F-12K medium containing 10% FBS. Parental and derivative mouse bladder cancer cell line MBT2 cells were maintained in ATCC-formulated Eagle’s Minimum Essential medium containing 10% FBS. Lenti-X-293T cells (Clontech Laboratories, Inc., cat. # 632180), a cell line for lentiviral packaging, was maintained in Dulbecco’s modified Eagle’s medium (DMEM, Invitrogen, cat. # 11965) containing 10% FBS and 4 mM l-glutamine.

### Generation of genetically engineered cell lines

RXRα^WT/MUT^ cDNAs were synthesized by Genewiz and cloned into the pInducer lentiviral vector. To overexpress hPPARγ, cDNA (GeneCopoeia, cat. # GC-Z0320) was procured and cloned into the pLenti6.3 lentiviral vector. Hairpin sequences to knockdown hPPARγ and RXRα were designed using the Broad Institute GPP web portal, synthesized by IDT and cloned into the pLKO-iKD-H1-puro lentiviral vector system. In all cases, lentivirus was produced by transfecting Lenti-X-293T cells with VSVG:delta R8.9:cloned vector constructs at a ratio of 1:4:4. Virus was harvested 2–3 days after transfection, filtered, and used to infect cell cultures in the presence of 8 μg/ml polybrene. In all cases, infected cells were selected with appropriate drugs in the media. RXRα^WT/MUT^ inducible overexpressing lines were generated by selection of respective lines in medium with 10% Tet-Free FBS with Geneticin for 2–3 weeks. Gene expression was induced by 3 day treatment with 100 ng/ml doxycycline (DOX). PPARγ constitutively overexpressing lines were generated by selection of respective lines in medium with 10% FBS plus blasticidin for 2–3 weeks. PPARγ knockdown lines were generated by selection of respective lines in medium with 10% Tet-free FBS with puromycin for 2–3 weeks. Stable cell lines infected with control vectors were generated to be used as negative controls for in vitro and in vivo experiments. All primer sequence information and antibiotic concentrations used to select the various derivative lines are itemized in Supplementary Table 5.

### Proliferation assays

Growth kinetic assay: 1000 cells were seeded in 96-well plates in media supplemented with 10% Tet-free FBS. For each cell line, cells were treated with 100 ng/ml DOX or without DOX. Growth rate for −/+DOX cells was measured every 2 or 3 days using CellTiter-Glo Luminescent Cell Viability Assay Reagent (Promega, cat. # G7573).

Long-term colony formation assays: 5000–7500 parental cells were seeded in six-well plates in media supplemented with 10% FBS. Compound was added the day after seeding and media/compound was replaced every 3 days. Viable colonies were stained after 2–3 week cultures. Following media aspiration, 0.5% crystal violet was added to the wells for about 1 h. Then crystal violet solution was removed and wells were carefully washed with H_2_O to remove residual staining solution. The plates were air-dried overnight prior to imaging.

### RNA extraction and quantitative real-time PCR

For assessing basal gene expression differences, parental lines were seeded for 24–48 h prior to sample collection, RXRα^WT/MUT^/shPPARγ lines (and respective vector controls) were cultured with 100 ng/ml DOX for 3 days prior to sample collection and PPARγ overexpressing lines (and respective vector controls) were seeded for 24–48 h prior to sample collection. Total RNA was extracted using the RNAeasy Mini Kit (QIAGEN, cat. # 74104) according to the manufacturer’s instructions. To assess changes in gene expression in response to test compounds, parental/engineered lines were cultured as described above prior to compound treatment at the described doses for 24 h prior to sample collection. For qPCR analysis, cDNA was made using the High Capacity cDNA Reverse Transcription Kit (Applied Biosystems, cat. # 4374966) and real-time PCR was performed in triplicates using TaqMan Gene Expression Master Mix (Applied Biosystems, cat. # 4369016) on an ABI ViiA 7 Real-Time PCR System. Expression levels were normalized to *GAPDH* expression.

### Sanger sequencing

Genomic DNA was isolated from HT-1197 parental cells using the Blood and Tissue DNAeasy Kit (Qiagen, cat. # 69581) according to the manufacturer’s instructions and sent to IDDEX for confirmation of the heterozygous RXRα^S427F^ mutation.

### Immunoblot analysis

Cells were lysed in loading buffer (Invitrogen, cat. # NP0007) containing protease inhibitor (Roche, cat. # 05892791001) and reducing reagent DTT (Invitrogen, cat. # NP0009), sonicated and subsequently boiled for 5 min. Approximately 20 μg of protein was loaded per lane and resolved by SDS polyacrylamide electrophoresis. Protein was transferred onto nitrocellulose membranes, blocked in 5% low-fat milk and probing was carried out overnight with antibodies to PPARγ (C26H12, 1:1000 dilution) (Cell signaling, cat. # 2435S), RXRα (D-20, 1:200 dilution) (Santa Cruz, cat. # SC-553), p65 (Santa Cruz, cat. # SC-8008, 1:1000 dilution), Vinculin (Sigma-Aldrich, cat. # V4505, 1:5000 dilution), and GAPDH (Sigma, cat. # G9545, 1:5000 dilution). Membranes were incubated with horseradish peroxidase (HRP)-conjugated anti-rabbit secondary antibody (Millipore, cat. # AP136P, 1:10,000 dilution) for 1 h and signal was developed using the enhanced chemiluminescence (ECL) method (GE Healthcare). Scans of the original western blots are shown in Supplementary Fig. 27.

### Human chemokine array

Conditioned media from 48 h incubation of sub-confluent cells in 2% FBS containing media was applied to the Human Inflammation Array C3 (Raybiotech, cat. # AAH-INF-3-8) following the manufacturer’s instructions.

### Biochemistry

The ligand binding domains of the receptors His-TEV-PPARγ (234-505), His-TEV-RXRα-WT (223-462) and His-TEV-RXRα-S427F (223-462) were cloned into pET28a (EMD Millipore) via Nco1 and EcoR1 restriction sites. Proteins were expressed in *Escherichia coli* overnight at 18 °C after induction with 0.5 mM IPTG at an OD_600_ of ~0.8. Soluble protein was purified by Ni-NTA chromatography followed by size exclusion chromatography with a 16/60 Sephacryl S-300 column equilibrated in 25 mM Tris-HCl, pH 8.0, 100 mM NaCl, 10% glycerol, and 1 mM TCEP. Peak fractions were pooled and flash frozen in liquid N_2_. For His-TEV-RXRα-WT, two peaks were observed in the elution profile. The predominant peak corresponded to the inactive tetramer and the minor peak was the monomer and these were pooled accordingly. To assess their stability, we reran these fractions on the sizing column after overnight incubation at 4 °C. The monomer and tetramer fractions appear to be stable under these conditions, consistent with previous reports^33^. The tetrameric RXRα is known to be inactive in the absence of ligand, so the monomer fraction was used for subsequent SPR and TR-FRET assays. The size exclusion profiles for PPARγ, RXRα-S427F, and heterodimer were further characterized analytically on a 10/300 Superdex 200 column. For this analysis, equivalent molar volumes were loaded and the heterodimer was formed by mixing the two proteins at a 1:1 molar ratio.

### SPR analysis

SPR measurements were performed using a Biacore T200 equipped with CM5 sensor chips (GE). The RXRα-WT and RXRα-S427F surfaces were prepared using standard amine-coupling procedures in 10 mM Na-acetate buffer pH 5.5. The resulting immobilization levels ranged from 750 to 950 resonance units with estimated surface activity of ~30%. Interactions with PPARγ were analyzed in dose response from 3 μM to 24 nM by twofold serial dilutions. The running buffer was 50 mM Tris pH 7.5, 150 mM NaCl, 1 mM TCEP, 0.005% P20 with 1 M sodium chloride as the regeneration buffer. The association phase was 60 s and the dissociation phase was 120 s. After reference and buffer signal subtraction the data were fit to a steady state binding model, a 1:1 kinetic model as well as a heteroanalyte kinetic model using Biacore T200 evaluation software (Supplementary Fig. 8). These data and models were then exported and replotted in GraphPad Prism to generate the publication quality figure.

### Crystallography

His-TEV-RXRα-S427F (223–462) and untagged PPARγ (234–505) were co-expressed in *E. coli* using a bicistronic construct cloned into pET-28a (EMD Millipore). Soluble protein was obtained by overnight induction at 18 °C using 0.5 mM IPTG. Cells were harvested and protein was purified using Ni-NTA chromatography followed by overnight TEV protease cleavage of the His-tag and a polishing subtractive Ni-NTA step to remove the His6-tagged TEV. The flow through was concentrated and injected on a 16/60 Sephacryl S-300 column equilibrated in storage buffer (20 mM HEPES 7.5, 350 mM ammonium acetate, 1 mM EDTA)^35^. Peak fractions were pooled and concentrated to ~18 mg/ml and flash frozen in liquid N_2_. For crystallization, protein was formulated at 10 mg/ml (172 μM) in storage buffer and incubated with 9-cis-retinoic acid and rosiglitazone (860 μM each with final DMSO 5.7%), SRC-1 peptide (Ac-CPSSHSSLTERHKILHRLLQEGSPS-amide) (516 μM), and TCEP (2 mM). Crystals grew in the dark at room temperature from 0.5 + 0.5 μl sitting drops equilibrated over a reservoir containing 20–24% PEG3350 and 0.02 M Na citrate. Crystals were frozen in reservoir solution supplemented with the agonist ligands and 20% ethylene glycol. Data were collected at the Advanced Photon Source, LS-CAT 21-ID-F (Supplementary Table 1). The structure was solved by molecular replacement using MOLREP^59^ and refined using Refmac^60^ with ligand coordinates generated using JLigand^61^. Coordinates have been deposited in the Protein Data Bank with 5JI0.

### Modeling the functional consequence of RXRα^S427F/Y^

The helix–helix interaction mediated by Helix-11 is one of the main structural motifs in the dimer interface. Alignment of the C-terminal Helix-11 and -12 from available RXRα heterodimer crystal structures shows that S427 of RXRα is located at a central pivotal point where the two interacting helices start to diverge (Supplementary Figs. 5 and 6). In these structures, the central residues (*n*) interact with each other and with the third residue downstream (*n* + 3). If this third residue is small (LXR, PXR, RARα, CAR, and TR), S427F could be accommodated and there would likely be little impact on heterodimerizaiton. If the *n* + 3 residue is bulky (FXR, RXR, and VDR), there would likely be a clash with S427F detrimental to heterodimerization. The partner PPARγ is exceptional for two reasons. First, the *n* + 3 residue is a small threonine which can accommodate the F427 mutation. Second, the C-terminal residue Y477 of PPARγ folds back toward the central residue of Helix-11. As a result, the S427F mutant of RXRα may form a π–π stacking pair with Y477 of PPARγ to promote or stabilize heterodimer formation. Superposition of structures was carried out using the Protein Structure Alignment function of Schrodinger software via Maestro interface.

### Cohort selection and TMA/microarray of bladder cancer

Characteristics of bladder cancer patients, enrolled from three institutions (Bern, Vancouver, Southampton), are summarized in Supplementary Table 4. The Ethics Committees of each institute approved this study and all patients consented to analysis of their tumor tissues. The Protocol numbers are as follows: Bern, Switzerland KEK-Be 219/2015; Vancouver, BC, Canada H09-01628; Southampton, UK, 10/H0405/99. All patients were diagnosed for muscle invasive bladder cancer by pre-chemotherapy transurethral resection (TURBT). They received thereafter at least three cycles of neoadjuvant chemotherapy and underwent cystectomy and pelvic lymph node dissection. For all tissue sampling, hematoxylin/eosin-stained sections were used to identify representative areas of tumor. Of chemotherapy-naïve TURBT specimens two punches were taken per patient for TMA construction^62^. For gene expression analysis, total RNA was extracted from TURBT specimens from a 1 mm diameter core punch by using RNeasy FFPE kit (Qiagen, Valencia, CA). After cDNA amplification and labeling (Ovation WTA FFPE system and Encore Biotin Module (NuGen, San Carlos, CA)) samples were hybridized to GeneChip Human Exon 1.0 ST oligonucleotide microarrays (Affymetrix, Santa Clara, CA) according to the manufacturer’s recommendations. Quality control was assessed by Affymetrix Power Tools packages and internally developed metrics^63^. The SCAN algorithm was used to normalize and summarize the microarray data^64^. All array files for these cases are available from the National Center for Biotechnology Information’s Gene Expression Omnibus (NCBI-GEO) database (http://www.ncbi.nlm.nih.gov/geo/).

### Immunohistochemistry

For the Eisai cohort, FFPE samples used for staining were obtained from Proteogenex and banked at Eisai (Andover, MA) (Supplementary Table 2). Five-micron sections were prepared from FFPE samples using a rotary microtome (Leica Microsystems, RM2255). Sections were placed on the charged slides and thoroughly dried on a warm plate at 35–37 °C overnight. Protocol “Bond HRP Detection for FFPE Tissue, mouse Ab_Ver 2” was used for IHC staining using anti-human CD8 mouse antibody (clone 4B11, Leica Microsystem, Cat #NCL-L-CD8-4B11, final concentration 0.285 µg/ml). Protocol “Bond HRP Detection for FFPE Tissue, Rabbit Ab_Ver 2” was used for IHC staining using anti-human PPARγ rabbit mAb (clone C26H12, Cell Signaling, cat. # 2435, final concentration 1 µg/ml). IHC staining was performed in a Leica BOND-RX Autostainer (Leica Microsystems), a Leica ST5020 Multistainer and Coverslipper CV5030-TS5025 (Leica Microsystems) according to the manufacturer’s recommendations. Slides were digitized using the Aperio ScanScope whole slide scanning system. Digitized slides were reviewed visually using the ImageScope software version 12.0.1.5027 and the results (positive or negative staining in each sample) were compared for two duplicate runs. Staining patterns were also evaluated and compared for two duplicate runs. Informed consent was obtained from all patients by the Russian Oncological Research Center n.a. N.N. Blokhin Rams Ethics Committee.

For the bladder cancer meta-dataset, freshly cut tissue sections/TMAs were used to determine the protein expression of targets of interest. Each immunohistochemical assay was optimized and performed using the Ventana Discovery Ultra autostainer, with a species specific secondary antibody against the relevant primary antibodies, described below. In brief, tissue sections were incubated in Tris EDTA (cell conditioning 1: CC1) buffer, at 95 °C for 60 min to retrieve antigenicity, followed by incubation with primary antibody at 37 °C for 60 min (PPARγ, anti-rabbit, clone #2435, Cell Signaling Technology, 1:100 dilution; CD8, anti-mouse, NCL-L-CD8-4B11, Leica, 1:100 dilution). Bound primary antibodies were incubated with Ventana universal secondary antibody at 37 °C for 32 min and visualized using Ventana DAB Map detection kit.

Scoring was done by assigning four point scale system as well as percent composite. Descriptively, score 1 represents no staining/negative by any tumor cells, score 2 represents a weak stain, score 3 represents moderate intensity stain and score 4 with a strong intensity staining. The overall protein expression was determined by multiplying the intensity score with the percent positive staining. For CD8 staining, the positive cells were counted in each tissue core.

### Immunofluorescence microscopy

Cells were plated on cover slips in 24-well plates for 24 h, then fixed with 4% PFA, permeabilized with 0.5% triton, blocked in 3% BSA (PBS) and incubated with anti-p65 (Santa Cruz, cat. # SC-8008, 1:100 dilution) and anti-PPARγ (Cell Signaling, cat. # 2435S, 1:100 dilution) antibodies. Antigens were visualized using Alexa Fluor 488/594 conjugated antibodies (Thermo Fisher Scientific, 1:500 dilution). Nuclei were stained with DAPI Vectashield mounting media (Vector Laboratory). Images were acquired on Zeiss LSM-780 confocal microscope at ×40 magnification.

### Derivation of a PPARγ signature

Glmnet^65^ (Lasso and Elastic-Net Regularized Generalized Linear Models) is used to shrink the differential genes to a smaller subset that predicts PPARG expression. A differential gene list related to PPARG pathway activity is obtained from in vitro cell line model. This list went through the following steps to select the features that predict PPARG expression in TCGA BLCA cohort:

1. Filtering out low expressed genes: filter out genes with median log2 (TPM + 1) value across the patients <1
2. Filtering out less variable genes: filter out genes with MAD value of the expression <0.5
3. Adjust the base level expression to be zero: center the median of each gene to be zero
4. cv.glmnet from glmnet R package is used to shrink the given features into a smaller subset that predicts PPARG expression (*α* = 0.5 and standardize = FALSE)

### Discovery of GLMnet74

To translate this in vitro derived PPARγ signature into the clinical setting, a GLMnet classifier was generated as follows. The 170 genes from the in vitro PPARγ signature were used for non-hierarchical clustering of the samples of the provisional bladder TCGA (Supplementary Fig. 15a). The cluster with concordant gene expression, labeled in the vertical annotation bar was determined as PPARγ active, the remaining samples as PPARγ inactive. Strength of association of the 170 genes between the PPARγ active and inactive cluster was used for feature selection. The expression of the 74 selected features was central normalized in each patient and genes were modeled into GLMNET. Genomic tuning and modeling was performed by optimizing lambda and using 10-fold cross-validation (*α* = 1.0). Using leave one out cross-validation, model performance was controlled and optimized in this discovery set. The final model was locked (GLMnet74). In the independent data sets (Bladder cancer metadata set and MD Anderson) the expression of the 74 selected genes was central normalized in each patient before applying GLMnet74 to calculate the prediction scores.

### Discovery of immunesignature190

A PPARγ related, bladder cancer specific immune signature, was determined as follows: Of the Nanostring pan cancer immune panel (*n* = 771), genes used in the PPARG signature were removed (*n* = 36). 425/735 genes were significantly (holm corrected p-value) underexpressed in PPARγ active tumors. 190 genes with the strongest and lowest expression in PPARγ active tumors were selected. The relation of Immunesignature190 and PPARγ activity of bladder cancers was determined by Gene signature expression analysis in the Bladder cancer meta-dataset and MD Anderson data set.

### Differential gene analysis

Linear model for microarray (limma)^66^ was used for differential gene expression analysis. The input TPM was transformed by log2 (TPM + 1), where 1 is added for each TPM value to avoid the divergence of zero TPM.

### Gene set enrichment analysis

GSEA software was downloaded from broad institute through the following link: http://software.broadinstitute.org/gsea/downloads.jsp. “GseaPreranked” tool was used with the ranked list from the differential gene analysis^67^. The ranked list was created from the differential gene analysis with the *p* score defined as the following. The absolute value of the *p* score is defined as −log2 (adjusted *p* value), where the adjusted *p* value is calculated by limma from the differential gene analysis. The sign of the *p* score was defined as the sign of the log2-fold-change from limma. Biocarta, Kegg and Reactome gene sets were selected for further analysis (Supplementary Figs. 2 and 4). Enrichment analysis in the bladder cancer meta-dataset (Supplementary Fig. 16) and MD Anderson data set (Supplementary Fig. 17), were performed as follows: Genes were ranked with Signal2Noise and a weighted enrichment statistic was used to calculate Normalized Enrichment Scores, *p*-values and false detection rate. Enrichment of hallmark gene sets (MSigDB Collections) and Immunesignature190 were analyzed between PPARγ active and inactive tumors, respectively.

### Efficacy studies in MBT2 syngeneic tumor model

Animal experiments were conducted in accordance with internal animal care and use committee (IACUC) guidelines defined by the H3 Biomedicine IACUC. 5×10^5^ MBT2-RXRα^WT^ and -RXRα^S427F^ cells resuspended in 0.1 ml PBS were subcutaneously implanted in the right flank region of 6–8 week old female C3H mice. Sample size (*n* = 12 per group for all studies except *n* = 11 for WT groups in anti-PD1 study) was chosen based on previous checkpoint inhibitor experiments in the MBT2 model to ensure adequate statistical power. Mice were treated with 2 mg/ml DOX in drinking water (supplemented with 5% sucrose) from the day of implantation and DOX treatment was continued for the duration of the study. Prior to treatment, mice were assigned using a randomization block design. In brief, experimental animals were first divided into homogenous blocks based on tumor volume. Second, within each block, randomization of experimental animals to different groups was conducted. This randomization approach ensures that each animal has the same probability of being assigned to any given treatment group and therefore minimizes systematic error. Animal studies described herein were not blinded. Intraperitoneal biw dosing with PBS and 5 mg/kg anti-PD1 (CD279, Bio X Cell) or 5 mg/kg anti-CTLA4 (CD152, Bio X Cell) was initiated once the tumors reached ~ 100 mm^3^ and continued for the duration of the study. The body weights and tumor volumes were measured every 2–3 d. Tumor volumes were calculated based on the formula: Tumor volume = length × width^2^ × 0.5, where length = largest diameter of tumor (mm) and width = diameter perpendicular to length (mm). Data are expressed as the mean ± SEM for tumor volume. All doses and regimens were well tolerated. The differences in tumor volume during the study period between the PBS and anti-PD1 or anti-CTLA4 treatment groups were analyzed by a one-way analysis of variance (ANOVA) followed by the Tukey post-hoc test using the endpoint measurements. One mouse was excluded from WT group in anti-PD1 study because the tumor began to spontaneously regress while all other tumors grew 10–30 fold during the course of the study. Statistical analyses were performed using the GraphPad Prism version 5.04 (GraphPad Software, La Jolla, CA).

### Quantitation of tumor infiltrating immune cells

MBT2-RXRα^WT^ and -RXRα^S427F^ cell implantation and DOX treatment was initiated as described above. Once tumors reached ~100 mm^3^, animals were randomized and dosed with PBS. Tumors were collected on the second day following the first dose (~300 mm^3^, *n* = 6) and FACS analysis was performed to assess number of infiltrating CD8+ T cells in the MBT2-RXRα^WT^ and -RXRα^S427F^ tumors. In brief, tumors were collected, depleted of blood vessels/necrotic/calcified tissue and dissociated with Collagenase B/DNAase I followed by mechanical disruption into single cell suspensions. One million cells were transferred into FACS tubes followed by addition of CD3-PE, CD4-PerCP, CD8a-FITC and FoxP3-APC antibodies. One million cells were transferred into FACS tubes followed by addition of CD11b-PE antibody. Incubation was performed in the dark for 30 min. Tubes were spun down at 1200 rpm for 5 min, supernatant was discarded and cells were resuspended in 2 ml PBS. Tubes were spun down again, supernatant discarded and cells were resuspended in 150 μl PBS and analyzed by FACS machine.

### Statistics

Appropriate statistical methods were performed as described in specific Method sections. *p* *<* 0.05 was considered as statistically significant.

### Data availability

All array files relating to the meta-dataset are available from the National Center for Biotechnology Information’s Gene Expression Omnibus (NCBI–GEO) database (http://www.ncbi.nlm.nih.gov/geo/) under the accession code GSE87304. The provisional TCGA Bladder Urothelial Carcinoma and the MD Anderson bladder cancer data set (GSE48075) data sets referenced during the study are available in a public repository from the Broad Institute Firehose Pipeline (http://gdac.broadinstitute.org; downloaded on January 10, 2016) and Gene Expression Omnibus (NCBI–GEO) (http://ncbi.nlm.nih.gov/geo) websites, respectively. The authors declare that all the other data supporting the findings of this study are available within the article and its supplementary information files and from the corresponding authors upon reasonable request.

## Electronic supplementary material

Supplementary Information
