# Author Correction: Evasion of immunosurveillance by genomic alterations of PPARγ/RXRα in bladder cancer

**DOI:** 10.1038/s41467-019-10666-3

**Published:** 2019-06-04

**Authors:** Manav Korpal, Xiaoling Puyang, Zhenhua Jeremy Wu, Roland Seiler, Craig Furman, Htoo Zarni Oo, Michael Seiler, Sean Irwin, Vanitha Subramanian, Jaya Julie Joshi, Chris K. Wang, Victoria Rimkunas, Davide Tortora, Hua Yang, Namita Kumar, Galina Kuznetsov, Mark Matijevic, Jesse Chow, Pavan Kumar, Jian Zou, Jacob Feala, Laura Corson, Ryan Henry, Anand Selvaraj, Allison Davis, Kristjan Bloudoff, James Douglas, Bernhard Kiss, Morgan Roberts, Ladan Fazli, Peter C. Black, Peter Fekkes, Peter G. Smith, Markus Warmuth, Lihua Yu, Ming-Hong Hao, Nicholas Larsen, Mads Daugaard, Ping Zhu

**Affiliations:** 1 H3 Biomedicine Inc., 300 Technology Square, Cambridge, MA 02139 USA; 2 Department of Urologic Sciences, University of British Columbia, Vancouver, BC Canada V5Z 1M9; 3 Vancouver Prostate Centre, Vancouver, BC Canada V6H 3Z6; 4 Eisai Inc., 4 Corporate Drive, Andover, MA 01810 USA; 5 Department of Urology, University Hospital of Southampton, Hampshire, SO16 6YD UK; 6 Department of Urology, University of Bern, Bern, CH-3010 Switzerland

**Keywords:** Cancer genomics, Immunosurveillance

Correction to: *Nature Communications* 10.1038/s41467-017-00147-w, published online 24 July 2017.

The original version of this Article contained an error in the spelling of the author Htoo Zarni Oo, which was incorrectly given as Htoo Z. Oo. This has now been corrected in both the PDF and HTML versions of the Article.

