# Supplementary material for: Evasion of immunosurveillance by genomic alterations of PPARγ/RXRα in bladder cancer
Source: Nat Commun. 2017 Jul 24;8:103. doi: 10.1038/s41467-017-00147-w (PMC5524640; doi:10.1038/s41467-017-00147-w)
Supplement: Supplementary file 1 — Supplementary Information [file 41467_2017_147_MOESM1_ESM.pdf]

File Name: Supplementary Information
Description: Supplementary Figures and Supplementary Tables

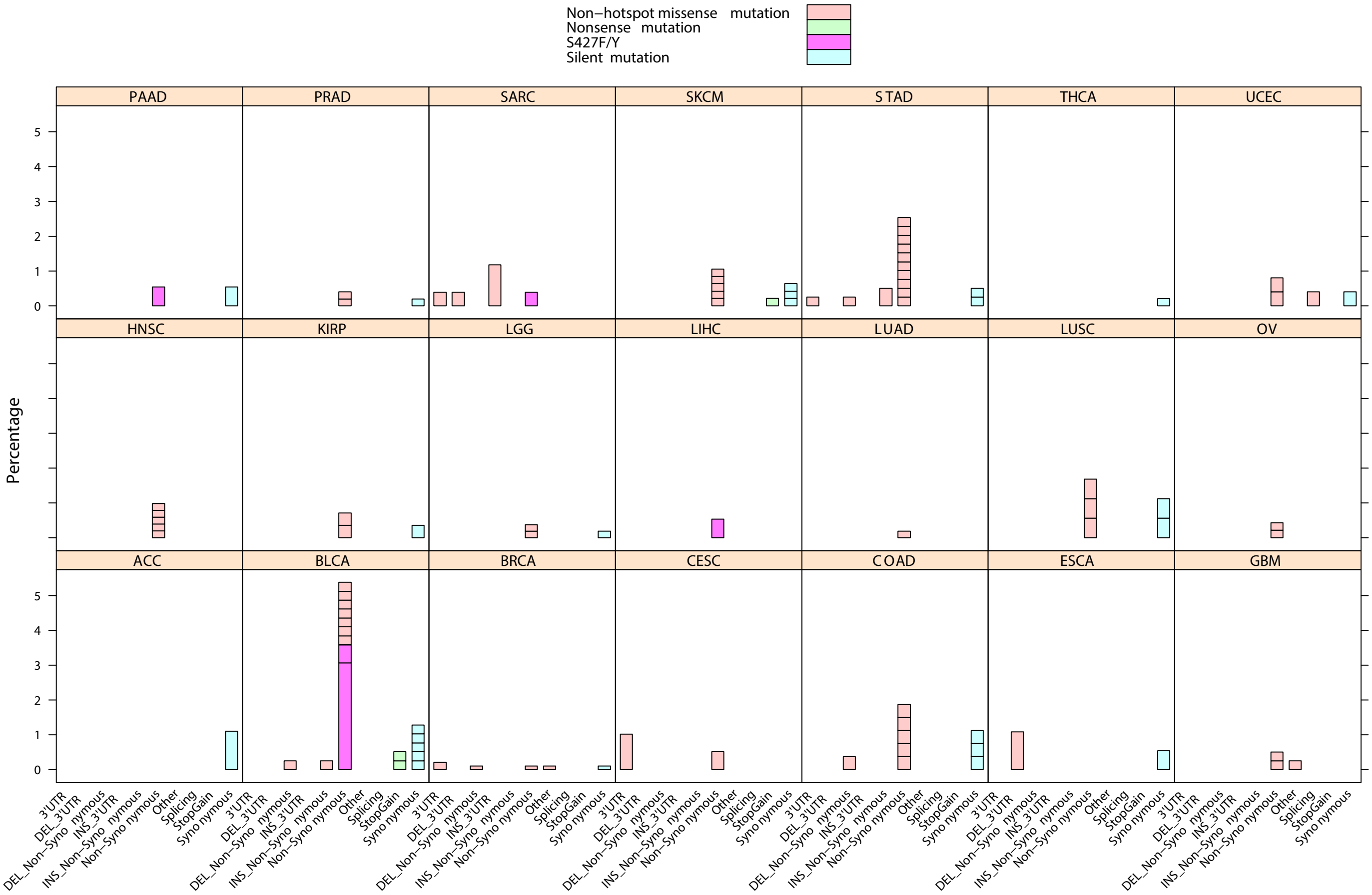

Supplementary Figure 1. Mutations of *RXRA* in different cancer types (TCGA). S427F/Y mutations are most frequent in bladder cancer compared with other cancer types.

Gene Set Name [# Genes (K)]	Description	# Genes in Overlap (k)	k/K	p-value	FDR q-value
REACTOME_METABOLISM_OF_LIPIDS_AND_LIPO IPOPROTEINS [478]	Genes involved in Metabolism of lipids and lipoproteins	40		9.15 e^{-16}	1.22 e^{-12}
REACTOME_FATTY_ACID_TRIACYLGLYCEROL_AN _AND_KETONE_BODY_METABOLISM [168]	Genes involved in Fatty acid, triacylglycerol, and ketone body metabolism	17		1.04 e^{-8}	6.89 e^{-6}
KEGG_PEROXISOME [78]	Peroxisome	11		1.4 e^{-7}	6.2 e^{-5}
KEGG_PPAR_SIGNALING_PATHWAY [69]	PPAR signaling pathway	10		3.99 e^{-7}	1.33 e^{-4}
REACTOME_PEROXISOMAL_LIPID_METABOLISM [21]	Genes involved in Peroxisomal lipid metabolism	6		1.35 e^{-6}	3.59 e^{-4}
REACTOME_GPCR_DOWNSTREAM_SIGNALING [805]	Genes involved in GPCR downstream signaling	35		1.64 e^{-6}	3.63 e^{-4}
REACTOME_TRIGLYCERIDE_BIOSYNTHESIS [38]	Genes involved in Triglyceride Biosynthesis	7		4.31 e^{-6}	8.18 e^{-4}
REACTOME_SIGNALING_BY_GPCR [920]	Genes involved in Signaling by GPCR	37		4.97 e^{-6}	8.26 e^{-4}
REACTOME_FATTY_ACYL_COA_BIOSYNTHESIS [18]	Genes involved in Fatty Acyl-CoA Biosynthesis	5		1.25 e^{-5}	1.84 e^{-3}
PID_LYSOPHOSPHOLIPID_PATHWAY [66]	LPA receptor mediated events	8		2.25 e^{-5}	2.74 e^{-3}
REACTOME_GENERIC_TRANSCRIPTION_PATHWAY [352]	Genes involved in Generic Transcription Pathway	19		2.26 e^{-5}	2.74 e^{-3}
KEGG_ENDOCYTOSIS [183]	Endocytosis	13		2.75 e^{-5}	3.05 e^{-3}
KEGG_TIGHT_JUNCTION [134]	Tight junction	11		3.01 e^{-5}	3.08 e^{-3}
REACTOME_SLC_MEDIATED_TRANSMEMBRANE_TR _TRANSPORT [241]	Genes involved in SLC-mediated transmembrane transport	15		3.24 e^{-5}	3.08 e^{-3}
REACTOME_PHOSPHOLIPID_METABOLISM [198]	Genes involved in Phospholipid metabolism	13		6.21 e^{-5}	5.51 e^{-3}
KEGG_OLFACTORY_TRANSDUCTION [389]	Olfactory transduction	19		8.63 e^{-5}	6.77 e^{-3}
REACTOME_SYNTHESIS_OF_VERY_LONG_CHAIN_ IN_FATTY_ACYL_COAS [14]	Genes involved in Synthesis of very long-chain fatty acyl-CoAs	4		8.65 e^{-5}	6.77 e^{-3}
KEGG_FATTY_ACID_METABOLISM [42]	Fatty acid metabolism	6		9.49 e^{-5}	7.01 e^{-3}
PID_IL8_CXCR1_PATHWAY [28]	IL8- and CXCR1-mediated signaling events	5		1.23 e^{-4}	8.64 e^{-3}
REACTOME_ACTIVATION_OF_CHAPERONE_GENES NES_BY_XBP1S [46]	Genes involved in Activation of Chaperone Genes by XBP1(S)	6		1.59 e^{-4}	1.06 e^{-2}
PID_CDC42_REG_PATHWAY [30]	Regulation of CDC42 activity	5		1.74 e^{-4}	1.1 e^{-2}
REACTOME_TRANSMEMBRANE_TRANSPORT_OF_SM _SMALL_MOLECULES [413]	Genes involved in Transmembrane transport of small molecules	19		1.86 e^{-4}	1.13 e^{-2}
REACTOME_SIGNALING_BY_RHO_GTPASES [113]	Genes involved in Signaling by Rho GTPases	9		1.97 e^{-4}	1.14 e^{-2}
KEGG_GAP_JUNCTION [90]	Gap junction	8		2.09 e^{-4}	1.16 e^{-2}
REACTOME_DAG_AND_IP3_SIGNALING [32]	Genes involved in DAG and IP3 signaling	5		2.39 e^{-4}	1.27 e^{-2}
KEGG_BUTANOATE_METABOLISM [34]	Butanoate metabolism	5		3.2 e^{-4}	1.64 e^{-2}
PID_ARF6_PATHWAY [35]	Arf6 signaling events	5		3.68 e^{-4}	1.73 e^{-2}
KEGG_CALCIUM_SIGNALING_PATHWAY [178]	Calcium signaling pathway	11		3.77 e^{-4}	1.73 e^{-2}
KEGG_INOSITOL_PHOSPHATE_METABOLISM [54]	Inositol phosphate metabolism	6		3.9 e^{-4}	1.73 e^{-2}
REACTOME_PHOSPHOLIPASE_C_MEDIATED_CASC ASCADE [54]	Genes involved in Phospholipase C-mediated cascade	6		3.9 e^{-4}	1.73 e^{-2}
KEGG_PHOSPHATIDYLINOSITOL_SIGNALING_SY _SYSTEM [76]	Phosphatidylinositol signaling system	7		4.16 e^{-4}	1.78 e^{-2}
REACTOME_PPARA_ACTIVATES_GENE_EXPRESSI SSION [104]	Genes involved in PPARA Activates Gene Expression	8		5.59 e^{-4}	2.29 e^{-2}
REACTOME_UNFOLDED_PROTEIN_RESPONSE [80]	Genes involved in Unfolded Protein Response	7		5.69 e^{-4}	2.29 e^{-2}
KEGG_T_CELL_RECEPTOR_SIGNALING_PATHWAY [108]	T cell receptor signaling pathway	8		7.18 e^{-4}	2.81 e^{-2}
BIOCARTA_EPONFKB_PATHWAY [11]	Erythropoietin mediated neuroprotection through NF-kB	3		8.33 e^{-4}	3.08 e^{-2}
REACTOME_REGULATION_OF_INSULIN_SECRETI ETION_BY_ACETYLCHOLINE [11]	Genes involved in Regulation of Insulin Secretion by Acetylcholine	3		8.33 e^{-4}	3.08 e^{-2}
REACTOME_OLFACTORY_SIGNALING_PATHWAY [328]	Genes involved in Olfactory Signaling Pathway	15		9.05 e^{-4}	3.25 e^{-2}
REACTOME_PLC_BETA_MEDIATED_EVENTS [43]	Genes involved in PLC beta mediated events	5		9.71 e^{-4}	3.4 e^{-2}
KEGG_VALINE_LEUCINE_AND_ISOLEUCINE_DEG DEGRADATION [44]	Valine, leucine and isoleucine degradation	5		1.08 e^{-3}	3.68 e^{-2}
PID_RHOA_REG_PATHWAY [46]	Regulation of RhoA activity	5		1.32 e^{-3}	4.4 e^{-2}

Supplementary Figure 2. Gene Set Enrichment Analysis (GSEA) for *RXRA*-S427F/Y vs. *RXRA*-WT bladder cancer (BLCA) samples from TCGA. PPAR pathway and lipid metabolism pathways that are regulated by PPARs are activated in bladder tumors harboring *RXRA*-S427F/Y.

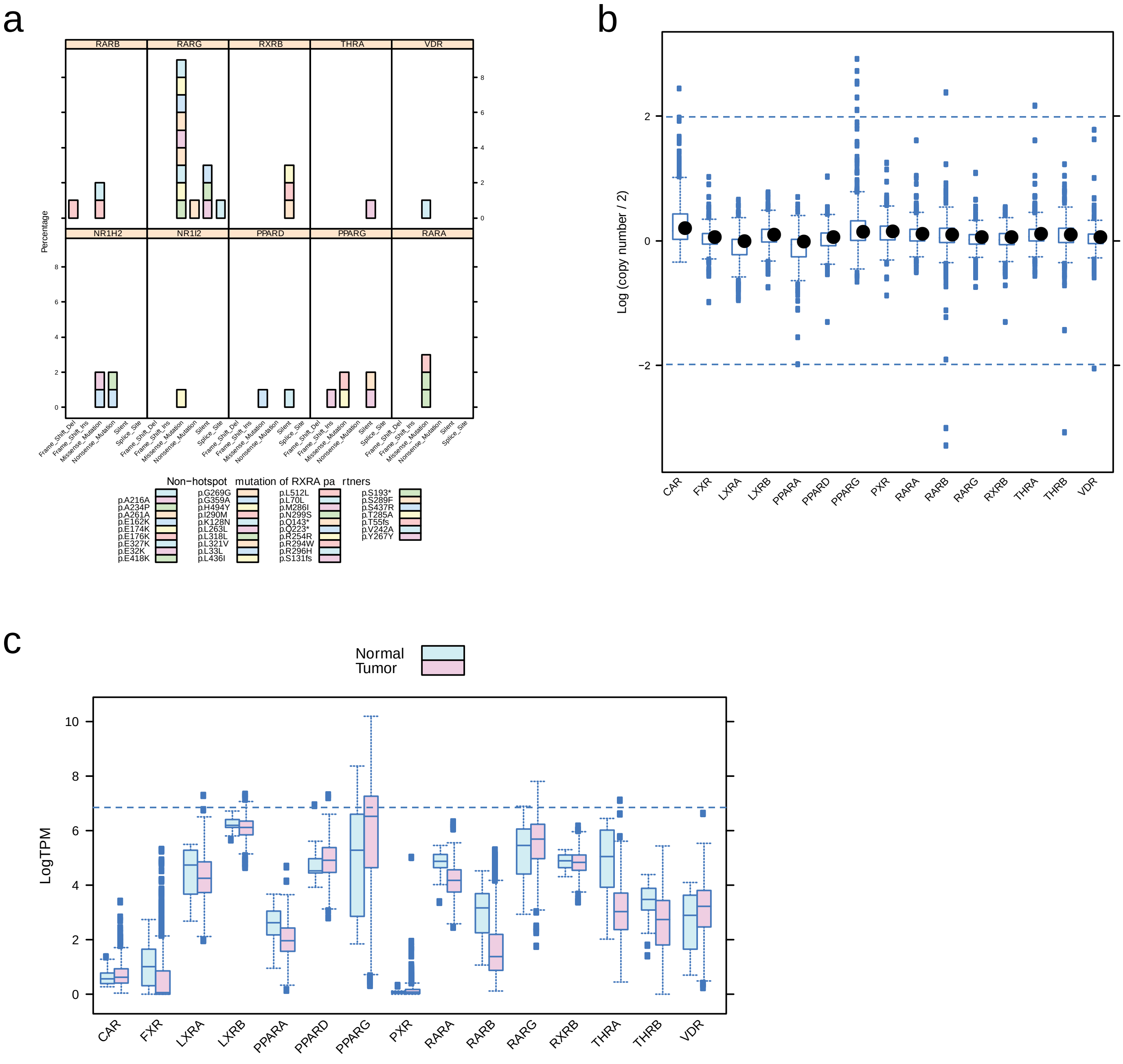

Supplementary Figure 3. Mutations, Copy Number Variation (CNV), and mRNA expression levels of RXRα heterodimerization partners in TCGA muscle-invasive bladder cancer (BLCA). (**a**) Mutations of partner genes of RXRα in TCGA cancers. None of these genes exhibit recurrent hotspot mutations. (**b**) CNV of partner genes of RXRα in TCGA muscle-invasive bladder cancer. *PPARG* is the only gene showing frequent high-level amplification (CN log2>2). (**c**) mRNA expression of partner genes of RXRα in TCGA muscle-invasive bladder cancer. A subset of tumors displayed overexpression of *PPARG*.

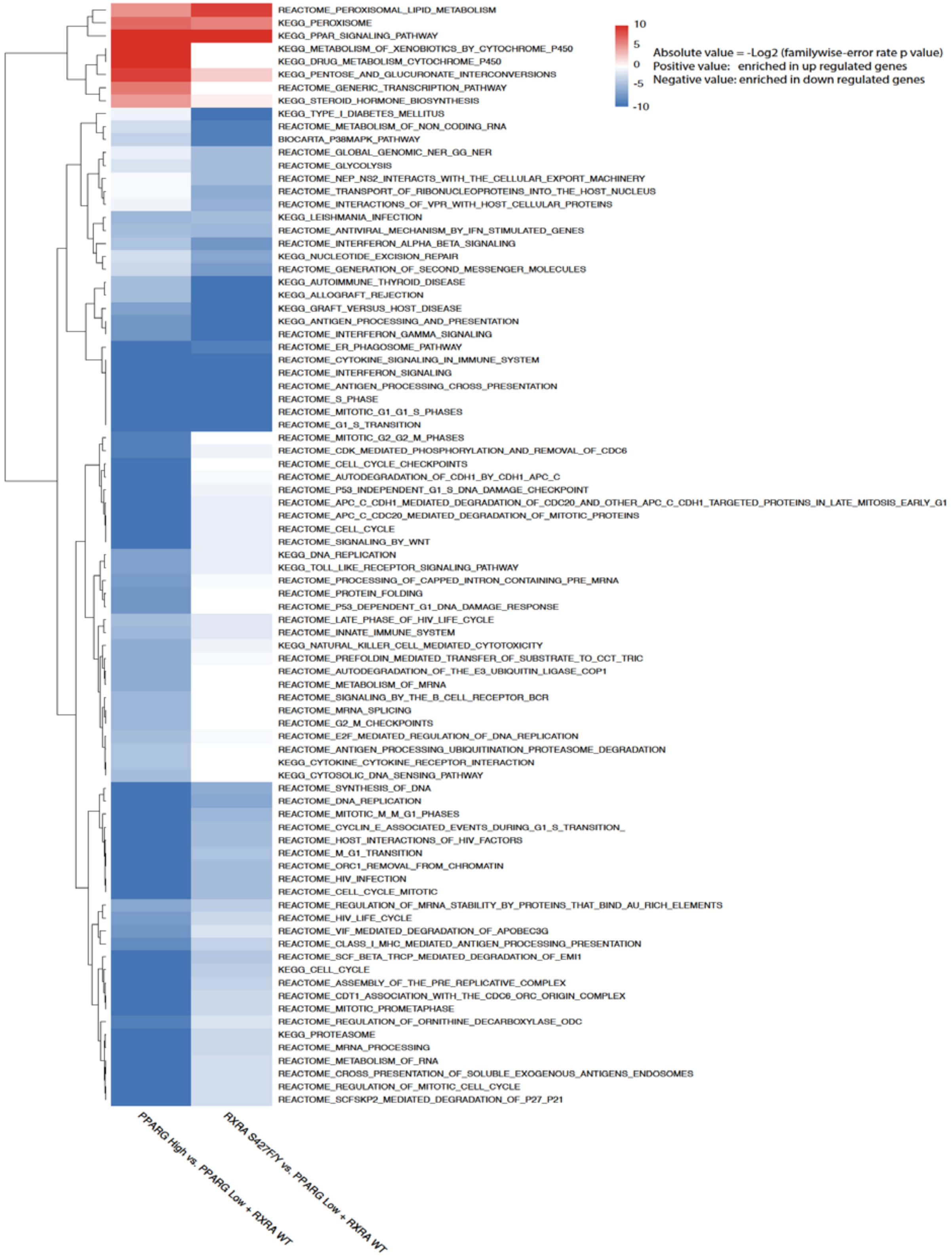

Supplementary Figure 4. Overlap of the activated or suppressed pathways in *RXRA*-S427F/Y and *PPARG*-overexpressed bladder tumors. TCGA muscle-invasive bladder cancer patients are split into 3 groups: RXRA S427F/Y hotspot mutant, PPARG overexpressed (log2 (TPM+1) > 7), double negative group (PPARG expression log2 (TPM + 1) < 4 and no RXRA S427F/Y hotspot mutation). Differential gene expression analysis is carried out for two pairs of comparisons. Comparison 1: RXRA S427F/Y hotspot mutants vs. double negative group. Comparison 2: PPARG high expressed vs. double negative group. GSEA analysis is used for both comparisons. The gene signatures that are significantly enriched in at least one of the two comparisons are selected to plot this heat map. The gradient scale represents –log2(familywise-error rate p value) from GSEA (http://software.broadinstitute.org/gsea/downloads.jsp).

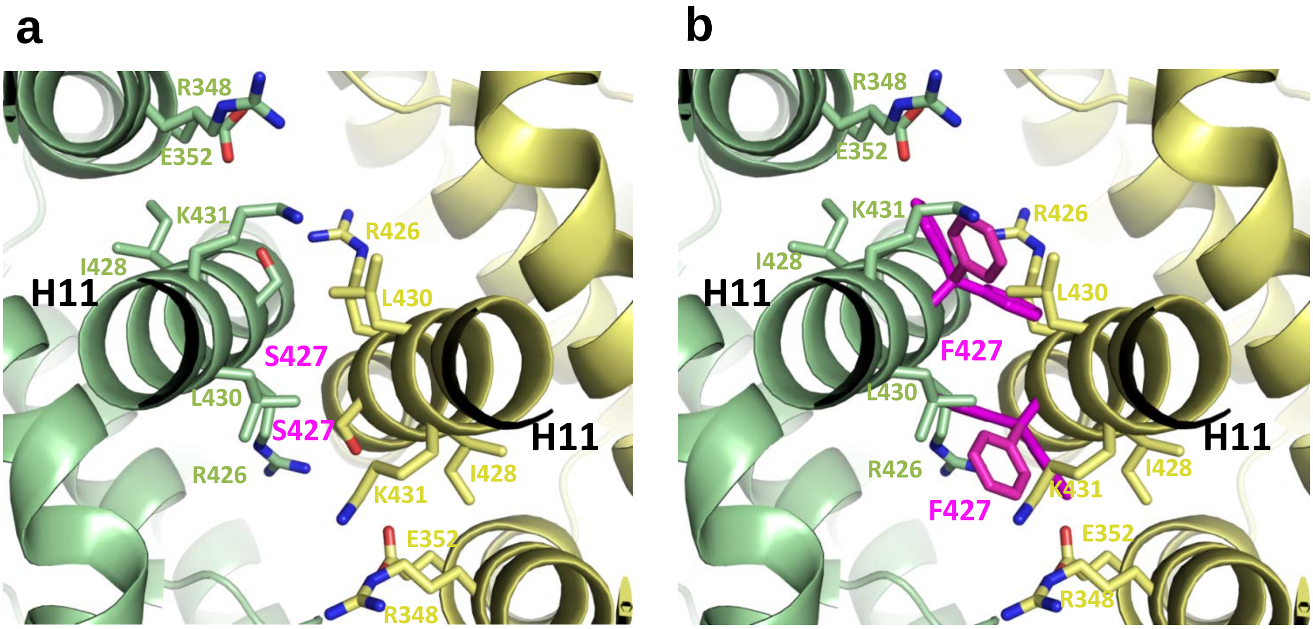

Supplementary Figure 5. The dimer interface formed in the RXRα^{WT} homotetramer structure (PDB-id 3R29). This view is along the two-fold symmetry axis with the two monomers colored green and yellow. (**a**) In the wild type structure, S427 nestles into a hydrophilic pocket at the dimer interface formed by the helix-helix motif mediated by helix 11. (**b**) The mutation S427F (magenta) has been modeled into the WT structure. Three different rotamers are illustrated, each of which results in clashes with neighboring residues. Side chain of S427F mutant was modeled based on the three known rotamer states of Phe residue in protein structures, i.e. CHI_1 = g+(60º), t(180º) and g-(-60º). This modeling helps explain the observation for why the S427F mutation disrupts formation of the tetramer and favors the monomer.

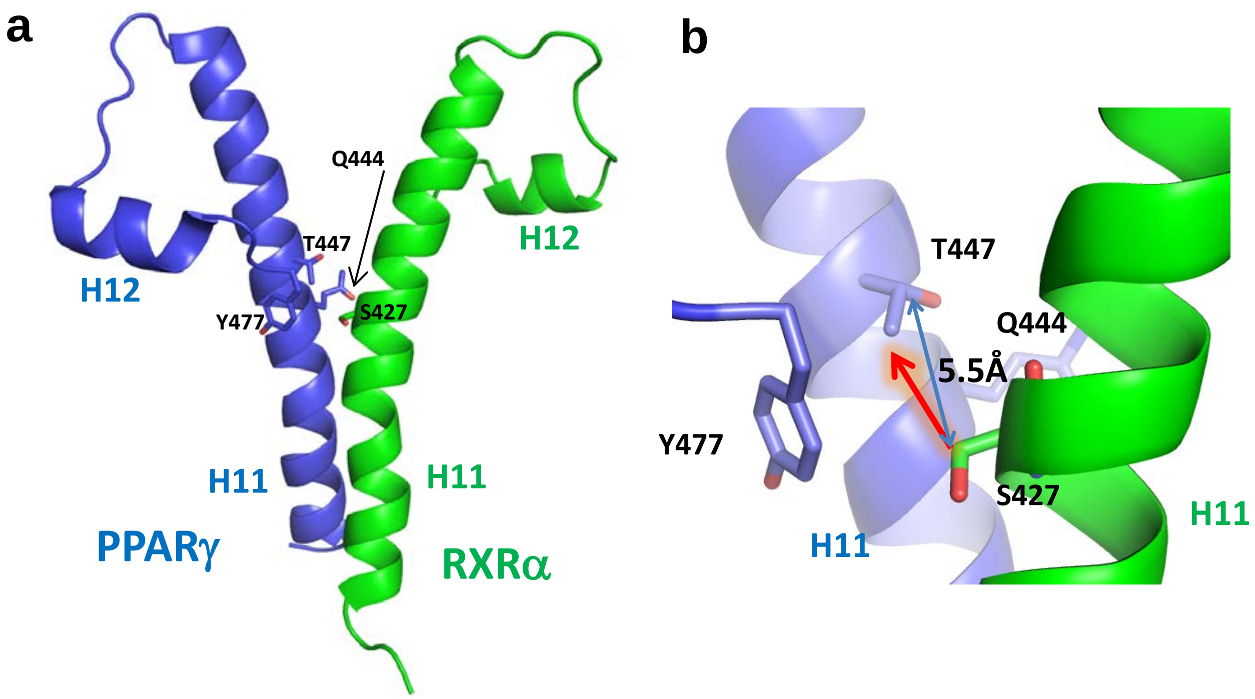

c

```
                                Helix-11                      Helix-12
                          hhhhhhhhhhhhhhhhhhhhhhhhhhhhh          hhhhhhh
RXRα     QPGRFAKLLLRLPALRSIGLKCLEHLFFFKLIG--DTPIDTFLMEMLEAPHQMT

                                n  3  6  9
PPARg    SSQLFAKLLQKMTDLRQIVTEHVQLLQVIKKT-ETDMSLHPLLQEIYKDLY
LXR      DRLMFPRMLMKLVSLRTLSSVHSEQVFALRLQ---DKKLPPLLSEIWDVHE
PXR      HRFLFLKIMAMLTELRSINAQHTQRLLRIQDI---HPFATPLMQELFGITGS
RAR      RPHMFPKMLMKITDLRSISAKGAERVITLKMEIP--GSMPPLIQEMLENSEGLDTLSG
CAR      SRFLYAKLMGLLAELRSINSAYSYEIHRIQGl---SAMMPPLLGEICS
TR       NIPHFWPKLLMKVTDLRMIGACHASRFLHMKVECPTELFPPLFLEVFEDQEV
FXR      NPQHFACLLGRLTELRTFNHHHAEMLMSWRVN---DHKFTPLLCEIWDVQ
VDR      SHLLYAKMIQKLADLRSLNEEHSKQYRCLSFQPECSMKLTPLVLEVFGNEIS
                          hhhhhhhhhhhhhhhhhhhhhhhhhhhhh          hhhhhhh
```

Supplementary Figure 6. Structural modeling and sequence alignment of the C-terminal Helix-11 and Helix-12 of RXRα and its heterodimer partners. (**a**) Global and (**b**) local views of the interface formed by Helix H11 in the PPARγ-RXRα heterodimer. Based on the packing, the S427F mutation could be accommodated in the PPARγ interface because the opposite sidechain T447 is small that can accommodate S427F side chain orientated in the red-arrow direction. There isn't room for two bulky sidechains at these positions in the interface. The PPARγ structural fragment was from x-ray crystal structure PDB-id 1FM9. (**c**) Sequence alignment of the C-terminal Helix-11 and Helix-12 of RXRα and its heterodimer partners. Helix-11s are the primary interacting motifs in formation of heterodimers between RXRα and other receptors. S427 of RXRα (bold font indicating the mutation position) is at the center of Helix-11. S427 pairs nearest the corresponding central residue **(n)** and **(n+3)**. When the n+3 residue is bulky (FXR and VDR), it would sterically interfere with a bulky S427F mutation in RXRα. Of all the RXRα dimer partners, only PPARs, including the gamma isoform, have an aromatic Tyr residue at the C-terminus that may form pi-stacking interaction with S427F mutation of RXRα. This is predicted to help stabilize the heterodimer between RXRα and PPARγ.

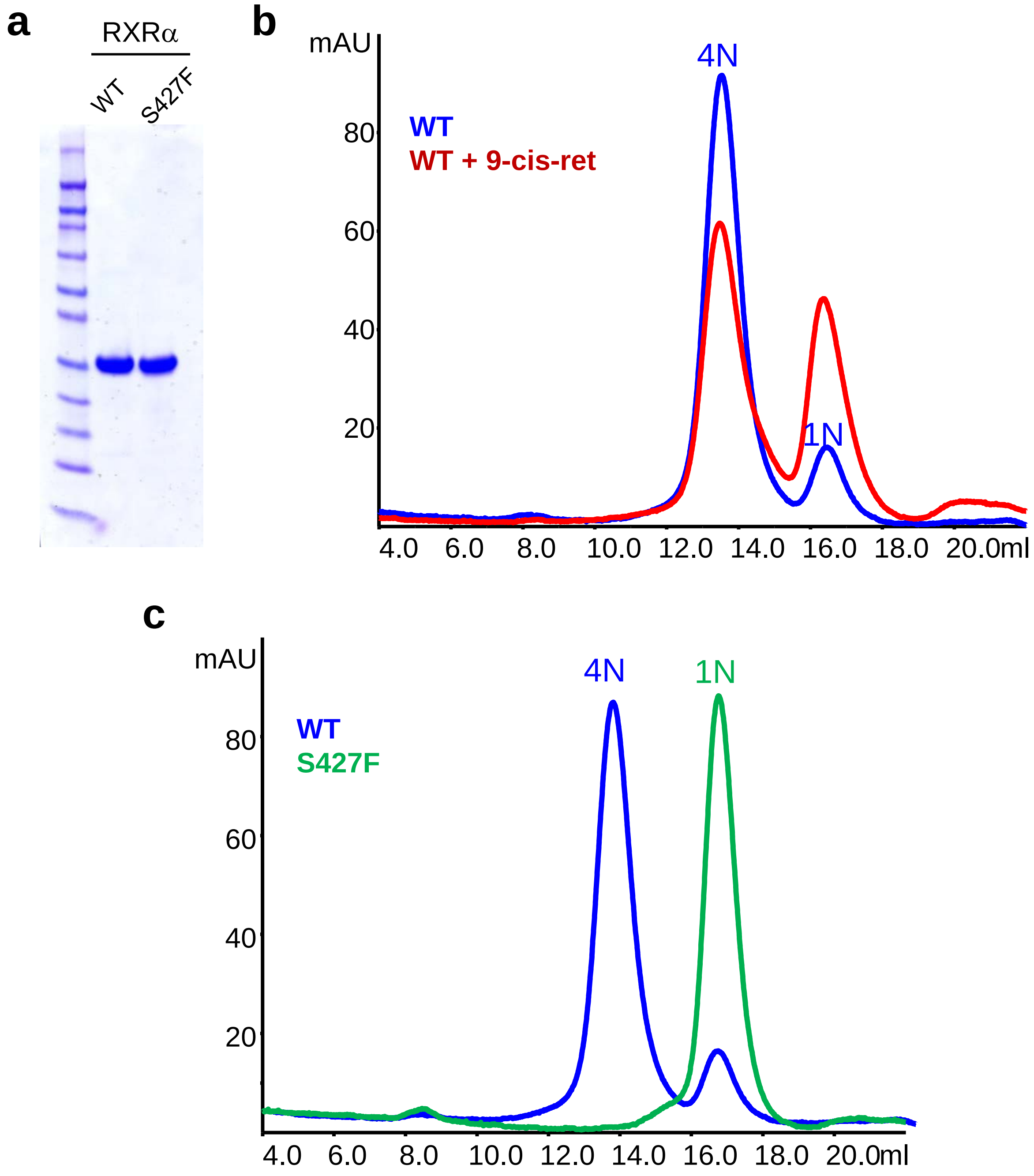

Supplementary Figure 7. Characterization of the recombinant RXRα LBD proteins. (**a**) Coomassie stained gel corresponding to the 4N WT peak and the 1N S427F mutant peak. (**b**) Elution profile of RXRα^{WT}. The WT receptor (blue trace) elutes predominantly as the homotetramer (4N) with a minor peak corresponding to the monomer (1N). Addition of ligand 9-cis-retinoic acid (red trace) shifts the equilibrium from tetramer to monomer. (**c**) The same elution profile for WT (blue trace) overlaid on the S427F mutant (green). The mutant profile is completely shifted to the monomer and is therefore primed for interaction with other nuclear receptors.

a

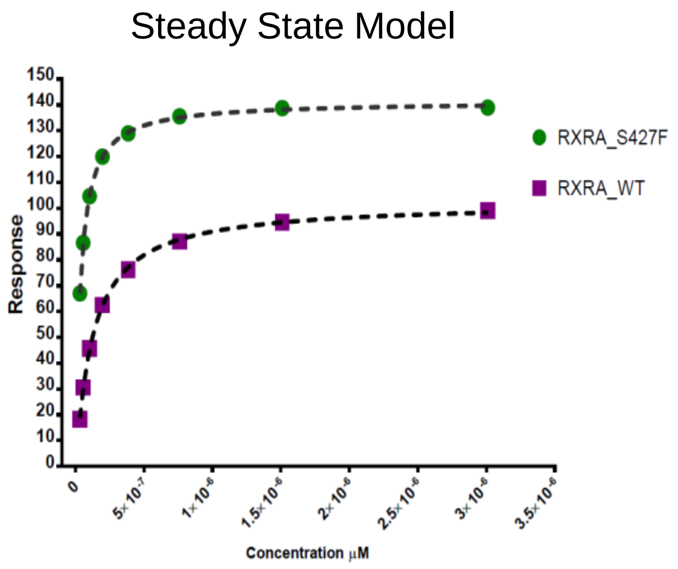

b

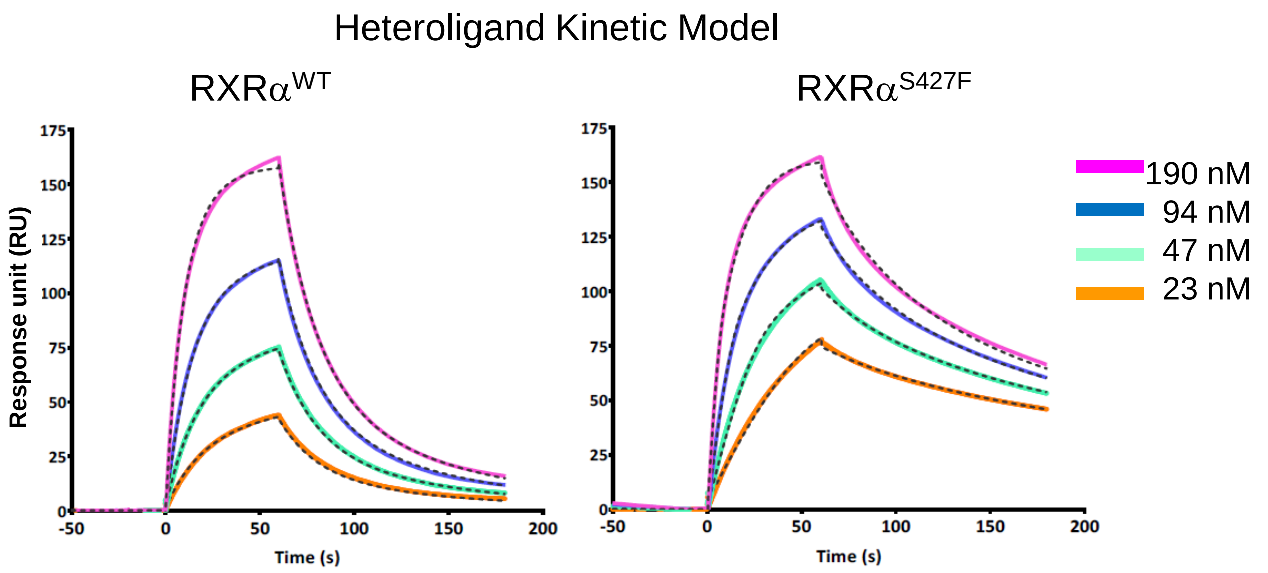

c

	Steady State model		1:1 kinetic model		Heteroligand kinetic model		
	K_D	Chi^2	K_D	Chi^2	K_{D1}	K_{D2}	Chi^2
**WT**	1.3E-7	0.6	6.4E-8	2.0	1.4E-7	3.9E-8	0.4
**S427F**	4.4E-8	0.4	6.7E-9	8.2	6.5E-8	1.6E-9	1.5

Supplementary Figure 8. SPR data analysis. (**a**) Steady state affinity model. The best fit was obtained using a report point after the end of injection. (**b**) Heteroligand kinetic model zoomed in on the lowest four concentrations. Concentrations indicated and modeled data shown as dashed lines. (**c**) Table of parameters with the dissociation constants (units of M) and the Chi^2 value for the quality of the fits. The steady state model and heteroligand kinetic models give the best fits to the data. All three models show that the mutant RXRα binds to PPARγ tighter than the wild type RXRα. Data were fit using the Biacore T200 Evaluation Software v3.0, and plotted using GraphPad Prism.

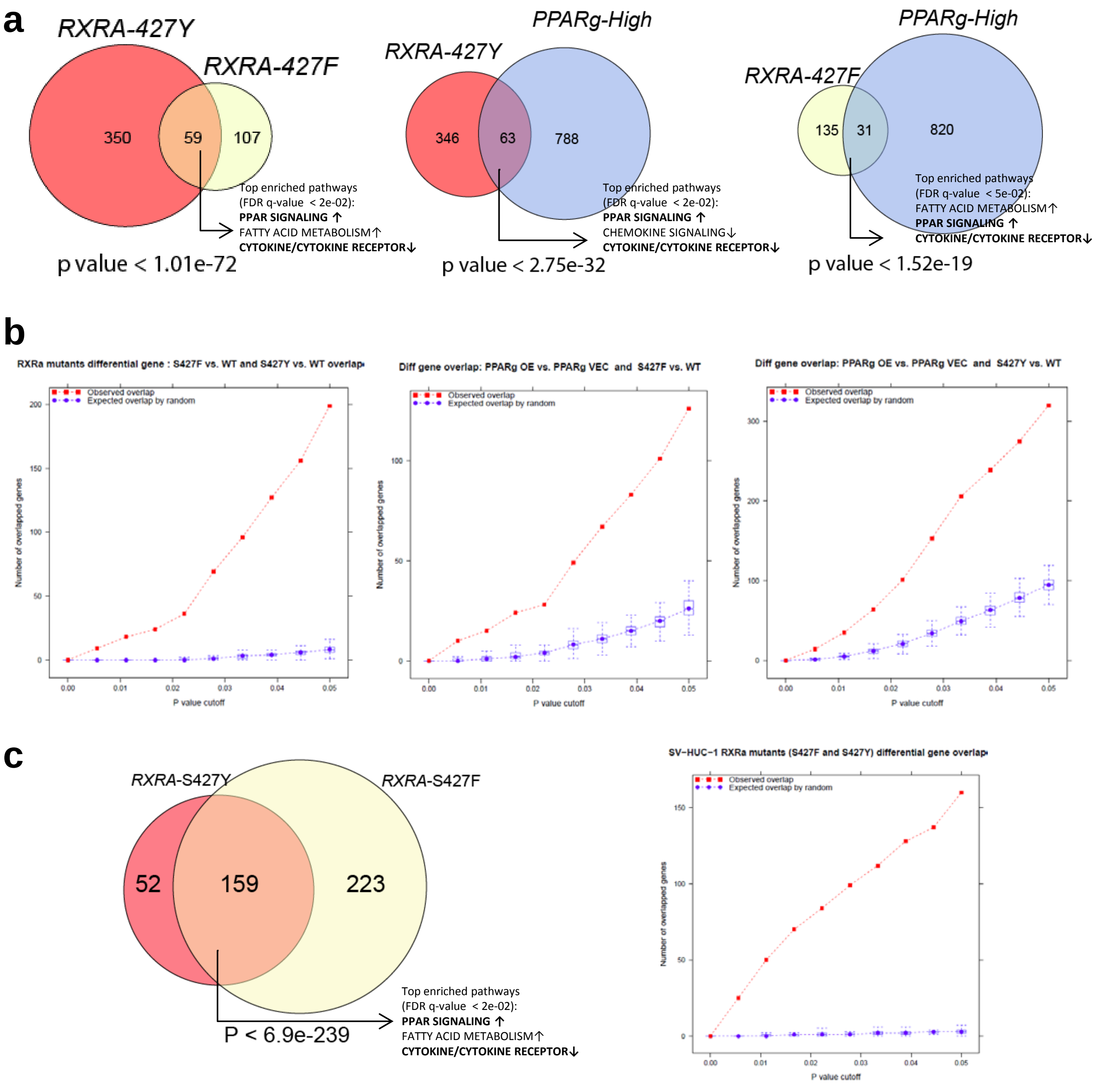

Supplementary Figure 9. Common transcriptional regulation by RXRα^{S427F}, RXRα^{S427Y} and PPARγ overexpression. (**a**) Significant differential gene overlap between RXRα^{S427F}, RXRα^{S427Y} and PPARγ-high. Venn diagrams show the number of genes differentially expressed (vs corresponding controls) following overexpression of RXRa^{S427F}, RXRa^{S427Y} or PPARγ in T24 cells. *p* value indicates the significance of the overlap compared to randomly expected overlap based on hypergeometric distribution. KEGG pathway enrichment analysis was performed for the overlap genes. Top upregulated (↑) and downregulated (↓) pathways were shown. (**b**) Significant differential gene overlap between RXRa^{S427F}, RXRa^{S427Y} and PPARg overexpression in T24 cells. (**c**) Significant differential gene overlap between RXRa^{S427F} and RXRa^{S427Y} in human immortalized normal bladder cells SV-HUC-1. For (**b**) and the right panel of (**c**), the red lines are the observed overlap curves from genes differentially expressed (vs corresponding controls). The blue lines are the randomly expected overlap curves between the two differential gene lists with error bars (obtained by random shuffling of the differential gene list). The error bars are the 95% confidence interval calculated based on bootstrap. *P* < 2e-16 for all plots analyzed by the two-way ANOVA.

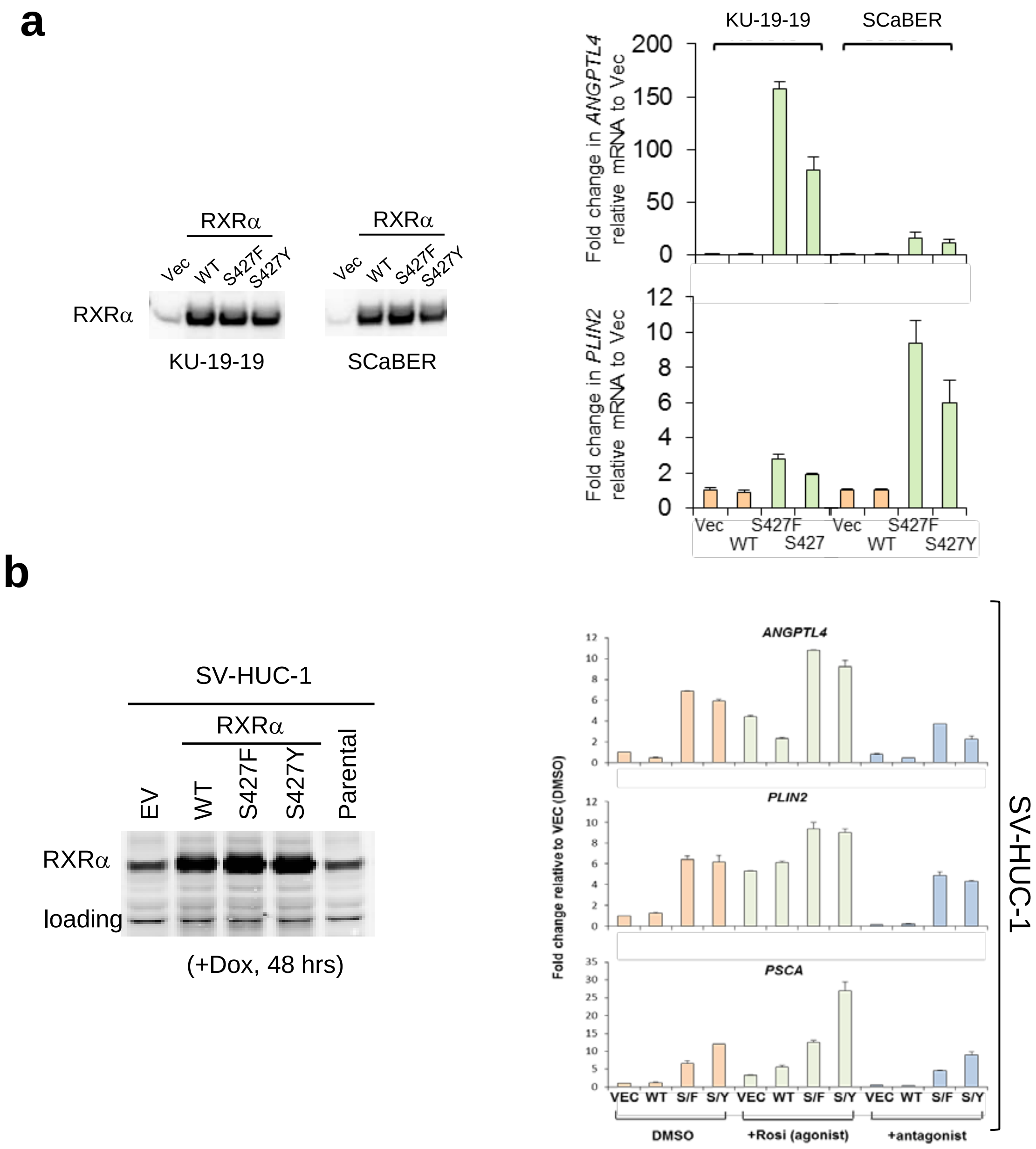

Supplementary Figure 10. RXRα$^{S427F/Y}$ can drive PPARγ transcriptional activity in a ligand-independent manner in several bladder cell lines. (**a**) *Left*, Western blot for RXRα in KU-19-19 and SCaBER bladder cancer cell lines engineered to overexpress RXRα^{WT}, RXRα^{S427F} and RXRα^{S427Y}. *Right*, RT-qPCR analysis of PPARγ target genes *ANGPTL4* and *PLIN2* in the vector control (Vec, beige bar), RXRα^{WT} (WT, beige bar), RXRα^{S427F} (S427F, green bar) and RXRα^{S427Y} (S427Y, green bar). (**b**) *Left*, Western blot of engineered SV-HUC-1 cell lines. *Middle*, RT-qPCR analysis for PPARγ target genes *ANGPTL4*, *PLIN2* and *PSCA* in the vector control (VEC, beige bar), RXRα^{WT} (WT, beige bar), RXRα^{S427F} (S427F, green bar) and RXRα^{S427Y} (S427Y, green bar) of SV-HUC-1 lines. *Right*, RT-qPCR analysis for *ANGPTL4*, *PLIN2* and *PSCA* in vector control (VEC), RXRα^{WT} (WT), RXRα^{S427F} (S/F) and RXRα^{S427Y} (S/Y) of SV-HUC-1 lines treated with DMSO (beige bars), PPARγ agonist rosiglitazone (Rosi, green bars) or PPARγ antagonist T0070907 (+antagonist) (blue bars) for ~16h. All data normalized to *GAPDH* and presented as fold change relative to VEC.

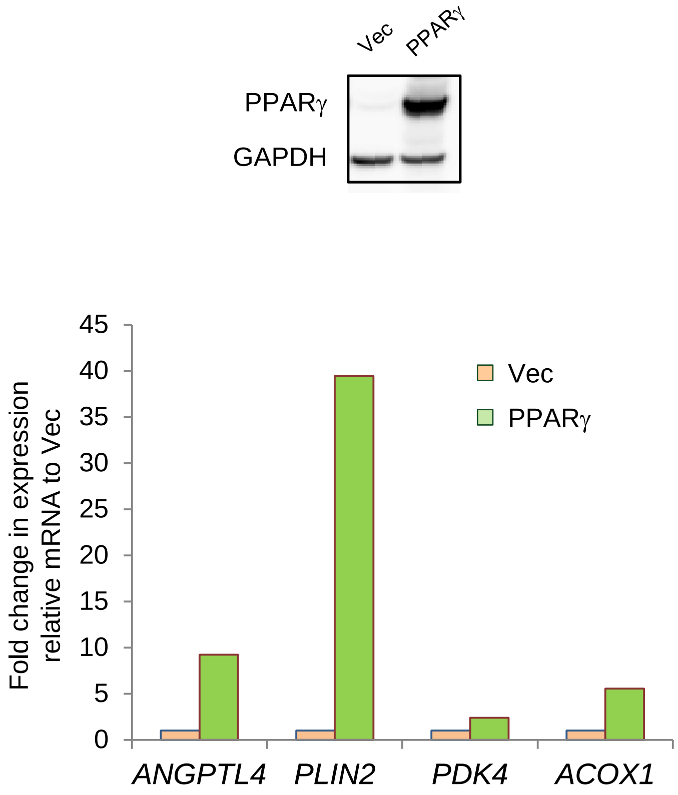

Supplementary Figure 11. Stable expression of PPARγ activates PPARγ-dependent transcription in bladder cancer cells. Stable PPARγ expression in SCaBER bladder cancer cells leads to ligand-independent activation of PPARγ signaling activity. *Upper*, Western blot for PPARγ and GAPDH in SCaBER bladder cancer cell line engineered to overexpress PPARγ-WT. *Lower*, RT-qPCR analysis of PPARγ target genes *ANGPTL4*, *PLIN2*, *PDK4* and *ACOX1* in the vector control (Vec, beige bar) and PPARγ overexpression (green bar) cells. All data normalized to *GAPDH* and presented as fold change relative to Vec.

a

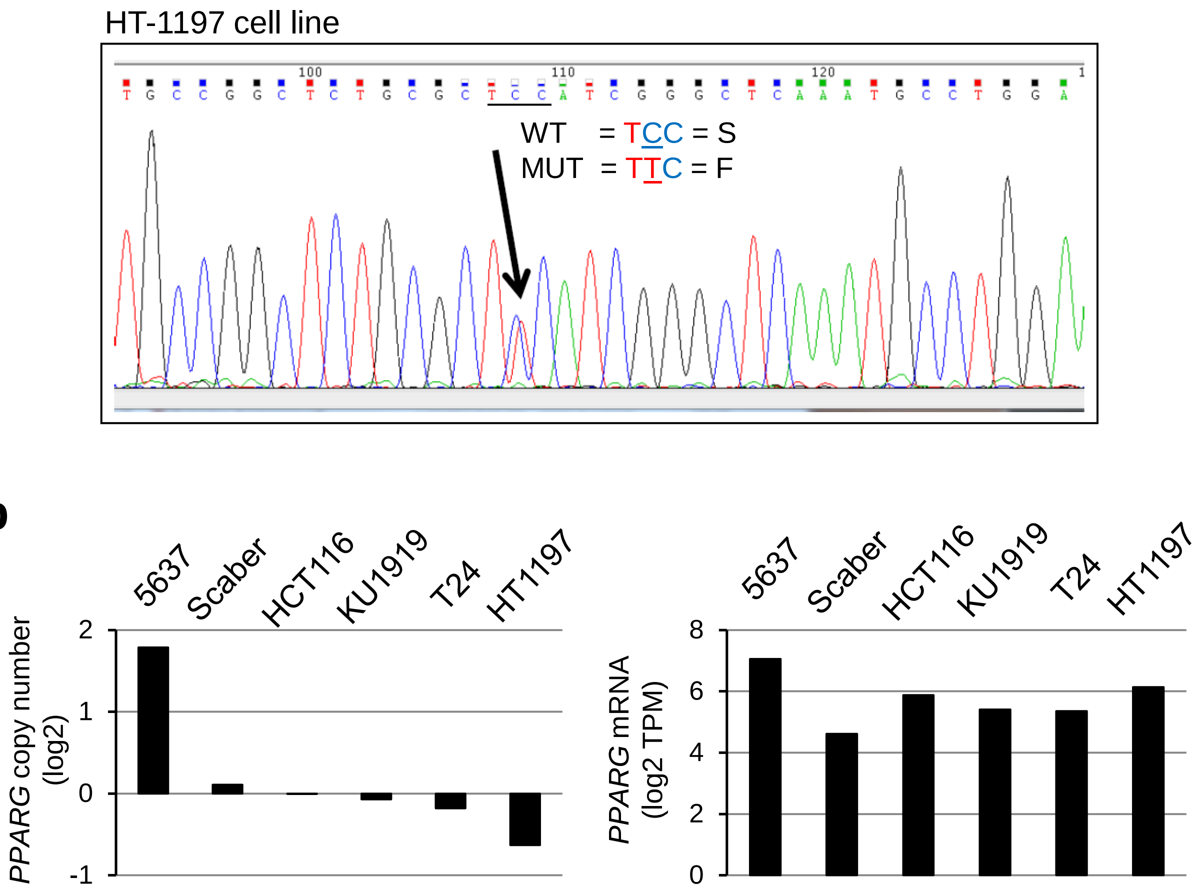

Supplementary Figure 12. Confirmation of *RXRA*-S427F in HT-1197and *PPARG* amplification in 5637 human bladder cancer cell lines. (**a**) Sanger sequencing trace file confirming S427F mutation in *RXRA* in HT-1197 human bladder cancer cell line. (**b**) Amplification and overexpression of *PPARG* in 5637 human bladder cancer cell line (CCLE dataset).

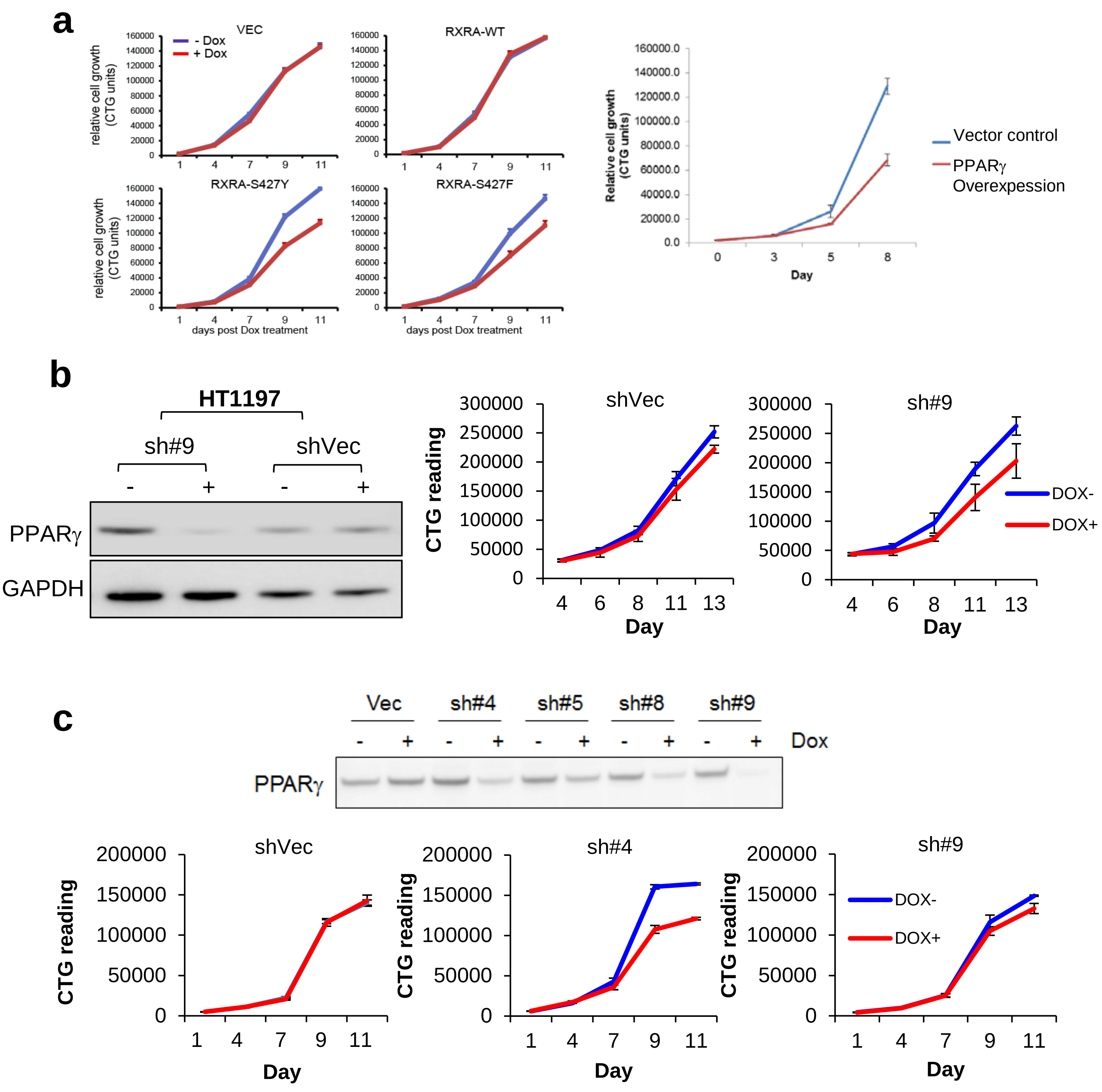

Supplementary Figure 13. ***In vitro* cell growth upon RXRα-mut expression or PPARγ knockdown in human bladder cancer lines.** (**a**) Inducible expression of RXRA-S427F or S427Y in T24 cells, or stable overexpression of PPARγ in SCaBER slightly impaired cell growth. **(b)** (*left*) Western blot for PPARγ in HT-1197 line following knockdown with shRNA #9 (sh#9). -, dox untreated; +, dox treated. -, dox untreated; +, dox treated. (*right*) Growth kinetics of control (shVec) and PPARγ knockdown line (sh#9). Blue curve, dox untreated; red curve, dox treated. (**c**) *Upper*, Western blot for PPARγ in 5637 line following knockdown with various shRNAs (sh#4, sh#5, sh#8 and sh#9). -, dox untreated; +, dox treated. *Lower,* Growth kinetics of control (shVec) and PPARγ knockdown lines (sh#4 and sh#9). Blue curve, dox untreated; red curve, dox treated.

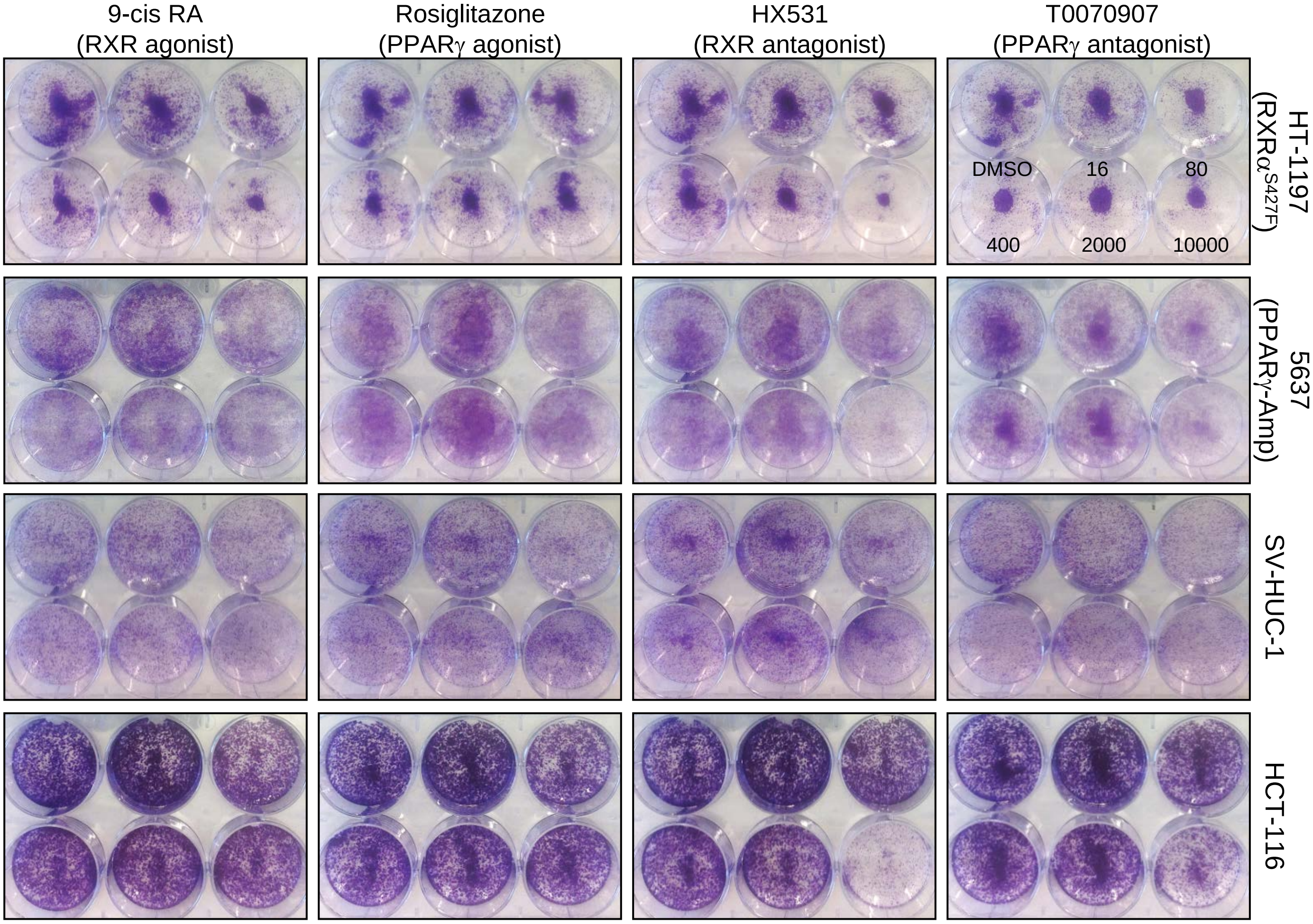

Supplementary Figure 14. Activity of ligands of RXRα or PPARγ on cell viability/growth. Long term colony formation assays for indicated lines treated with RXR agonist 9-cis retinoic acid (RA), PPARγ agonist rosiglitozone, RXRα antagonist HX531 and PPARγ antagonist T0070907 at the indicated concentrations (nM, labeled in HT1197 plate treated with T0070907). HCT-116 human colon carcinoma line was used as a control line.

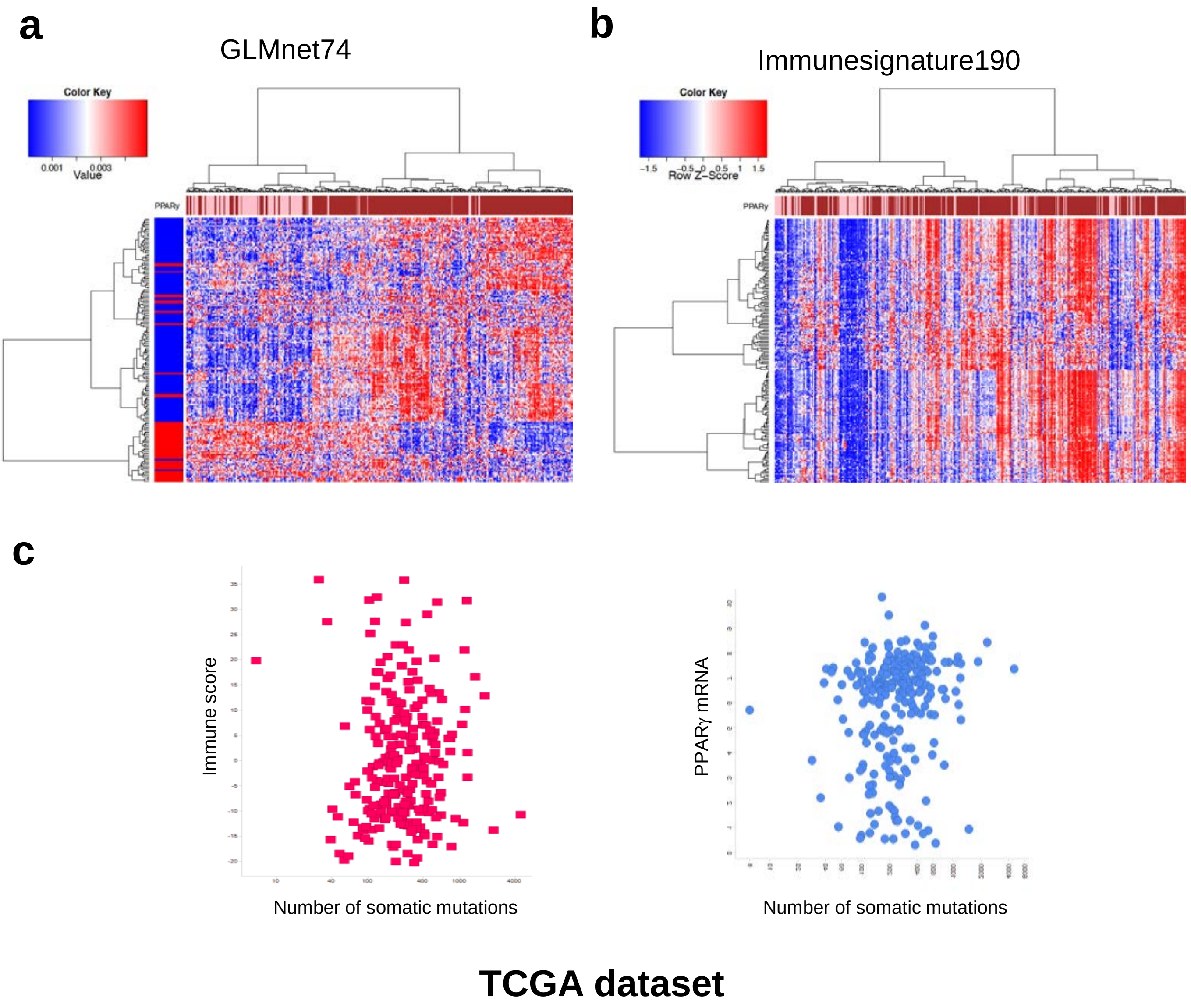

Supplementary Figure 15. TCGA muscle-invasive bladder tumor (MIBC) dataset demonstrates that PPARγ/RXRα pathway activity is negatively correlated with immune response. (**a**) GLMnet74 was applied to this dataset to assign the tumors to the PPARγ activity. This heatmap of the 171 PPARγ signature indicates the annotation to the PPARγ activity (pink: active, brown: inactive). (**b**) This enrichment plot shows the significant negative enrichment of the discovered immunesignature190 in PPARγ active tumors of this dataset. (**d**) Lack of correlaion (*left*) between somatic mutation load and immune score (infiltrates), and (*right*) between somatic mutation load and PPARγ expression.

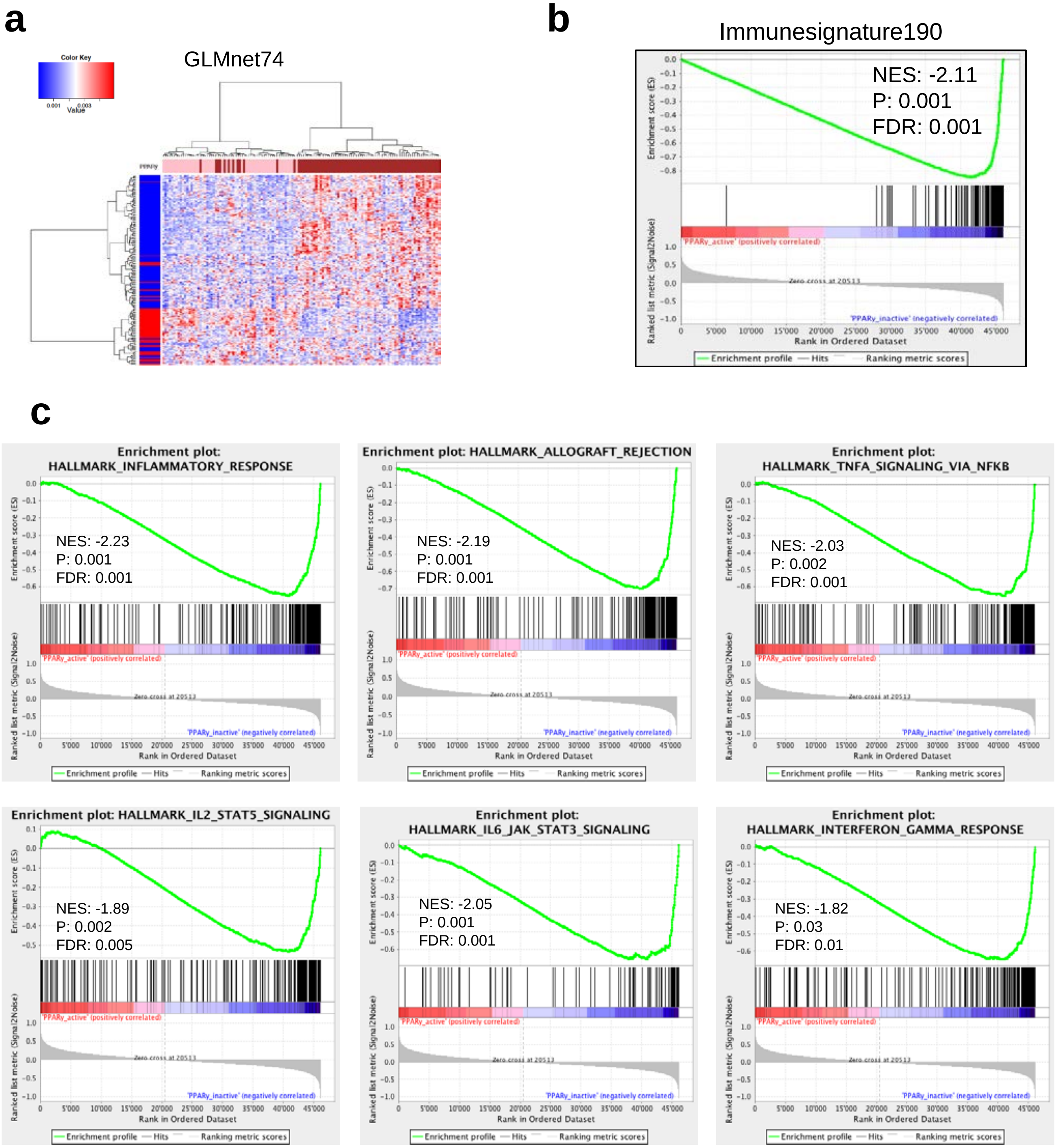

Bladder cancer meta-dataset

Supplementary Figure 16. Bladder cancer meta-dataset confirms that PPARγ/RXRα pathway activity is negatively correlated with immune response. (**a**) GLMnet74 was applied to this dataset (127 chemotherapy naive TURBT samples) to assign the tumors to the PPARγ activity. This heatmap of the 171 PPARγ signature indicates the annotation to the PPARγ activity (pink: active, brown: inactive). (**b**) This enrichment plot shows the significant negative enrichment of the discovered immunesignature190 in PPARγ active tumors of this dataset. (**c**) Hallmark gene signatures were compared between PPARγ active and inactive tumors. The second most negative enriched signature in PPARy active tumors was Hallmark_Inflammatory_Response (top left). Among the 14 most negative enriched signatures, 6 are related to immune response. The enrichment plots of those sigantures are shown here.

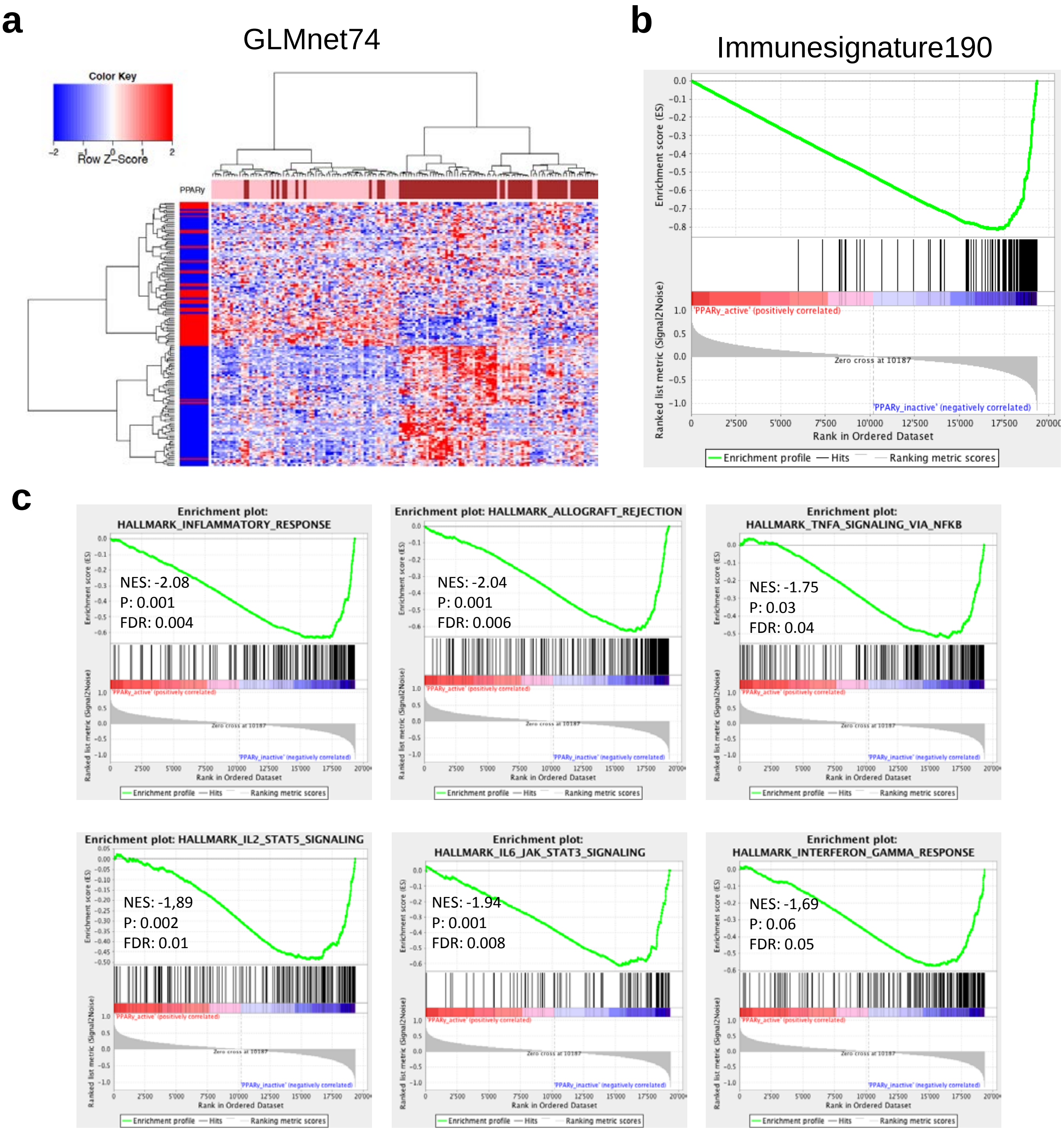

MD Anderson dataset

Supplementary Figure 17. MD Anderson bladder dataset (n=107) confirms that PPARγ/RXRα pathway activity is negatively correlated with immune response. (**a**) GLMnet74 was applied to this dataset to assign the tumors to the PPARγ activity. This heatmap of the 171 PPARγ signature indicates the annotation to the PPARγ activity (pink: active, brown: inactive). (**b**) This enrichment plot shows the significant negative enrichment of the discovered immunesignature190 in PPARγ active tumors of this dataset. (**c**) Hallmark gene signatures were compared between PPARγ active and inactive tumors. The second most negative enriched signature in PPARy active tumors was Hallmark_Inflammatory_Response (upper left). Among the 14 most negative enriched signatures, 6 are related to immune response. The enrichment plots of those sigantures are shown here.

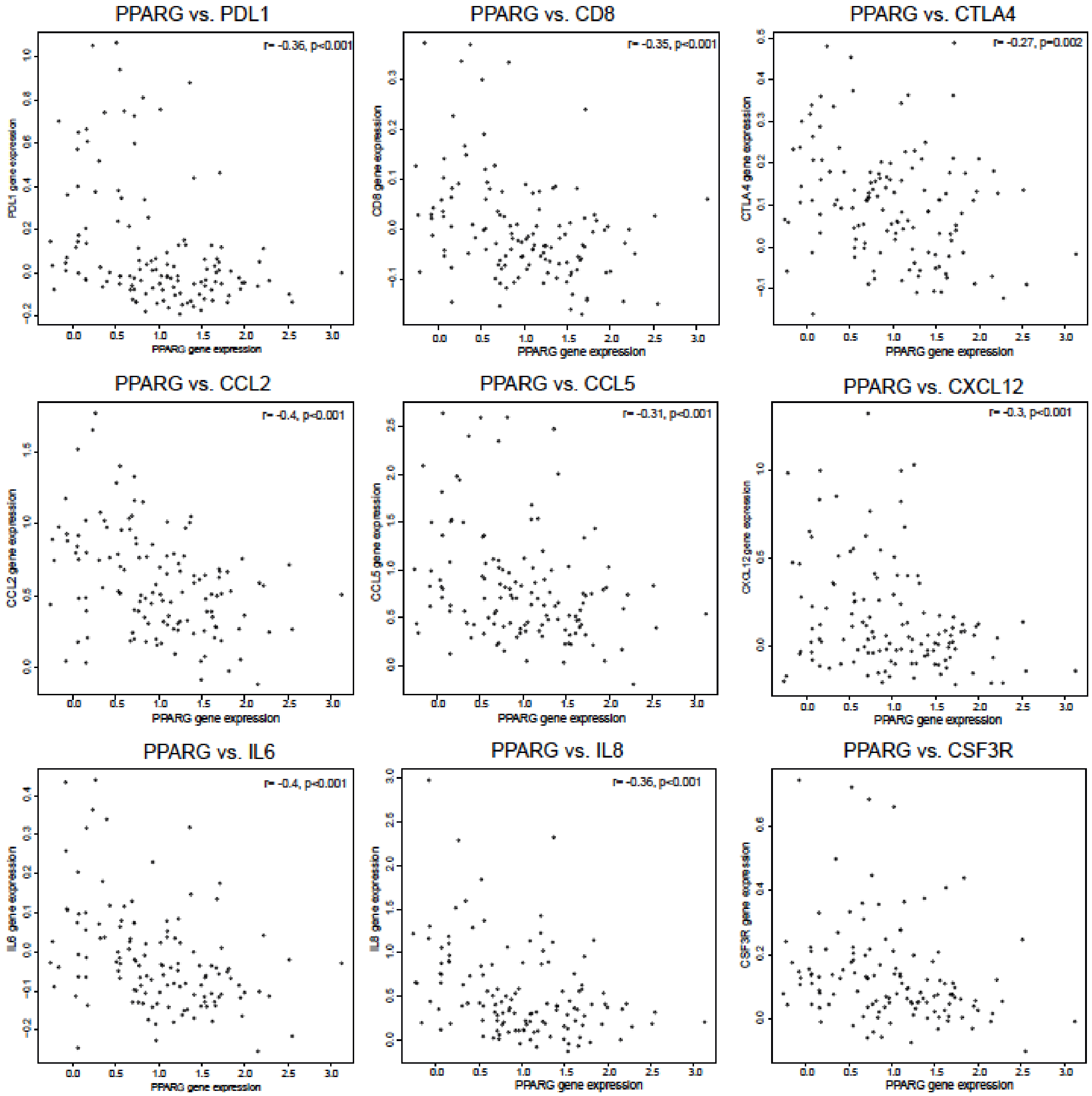

Supplementary Figure 18. *PPARG* expression in human bladder cancer is correlated with reduced T cell marker expression and secretion of inflammatory factors. Plots comparing the *PPARG* gene expression to the gene expression of a selection of T cell marker, immune checkpoint ligands/receptors, chemo- and cytokines in the Bladder cancer meta-dataset (127 chemotherapy naive TURBT bladder tumor samples). The gene expression of these immune markers are significantly negatively correlated with *PPARG*.

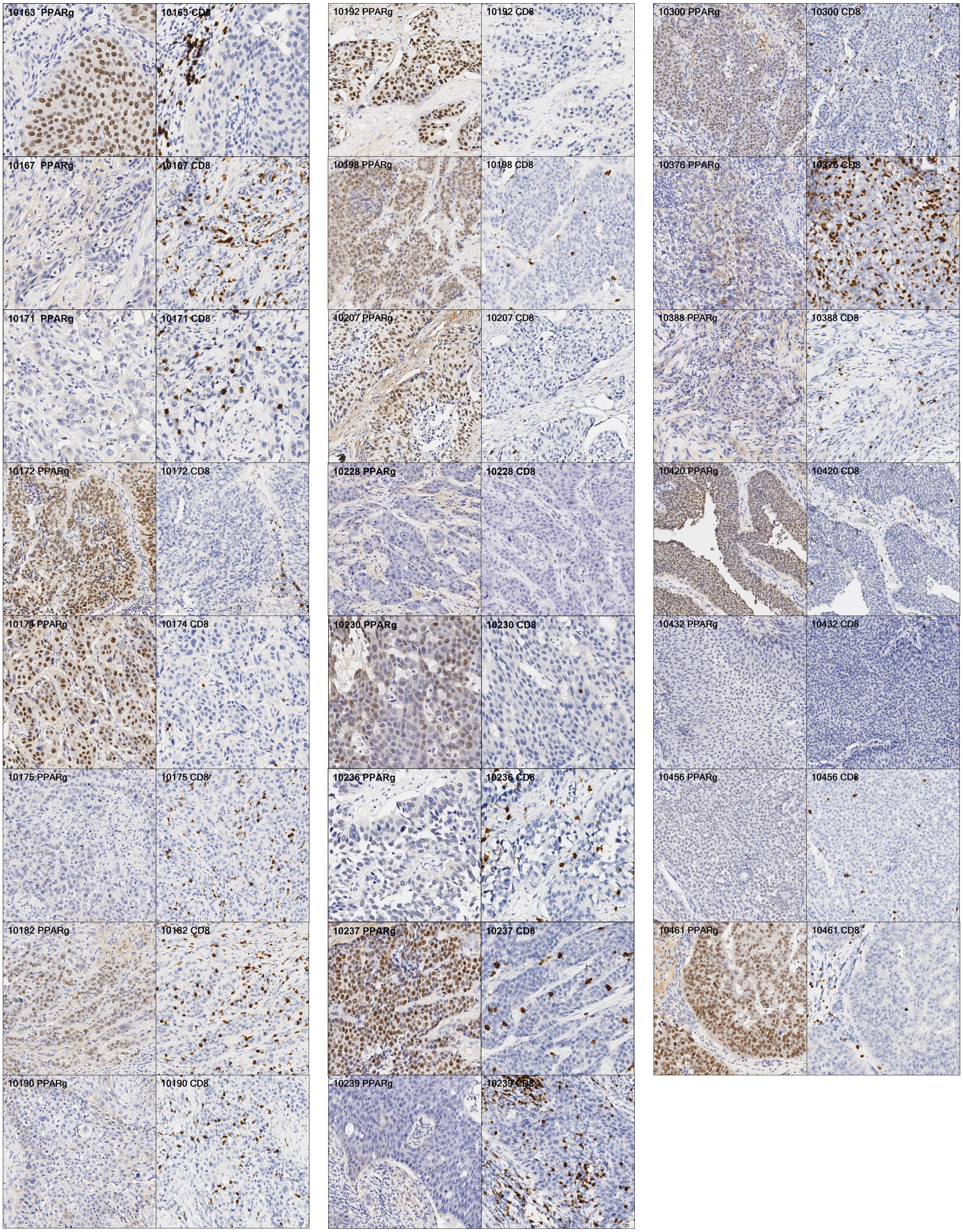

Supplementary Figure 19. Immunohistochemistry (IHC) staining of PPARγ and CD8 of the Eisai cohort of human bladder tumors. Patient ID and antibodies used for IHC are labeled at the upper-left corner of the images. The scores of IHC are summarized in Supplementary Table 3.

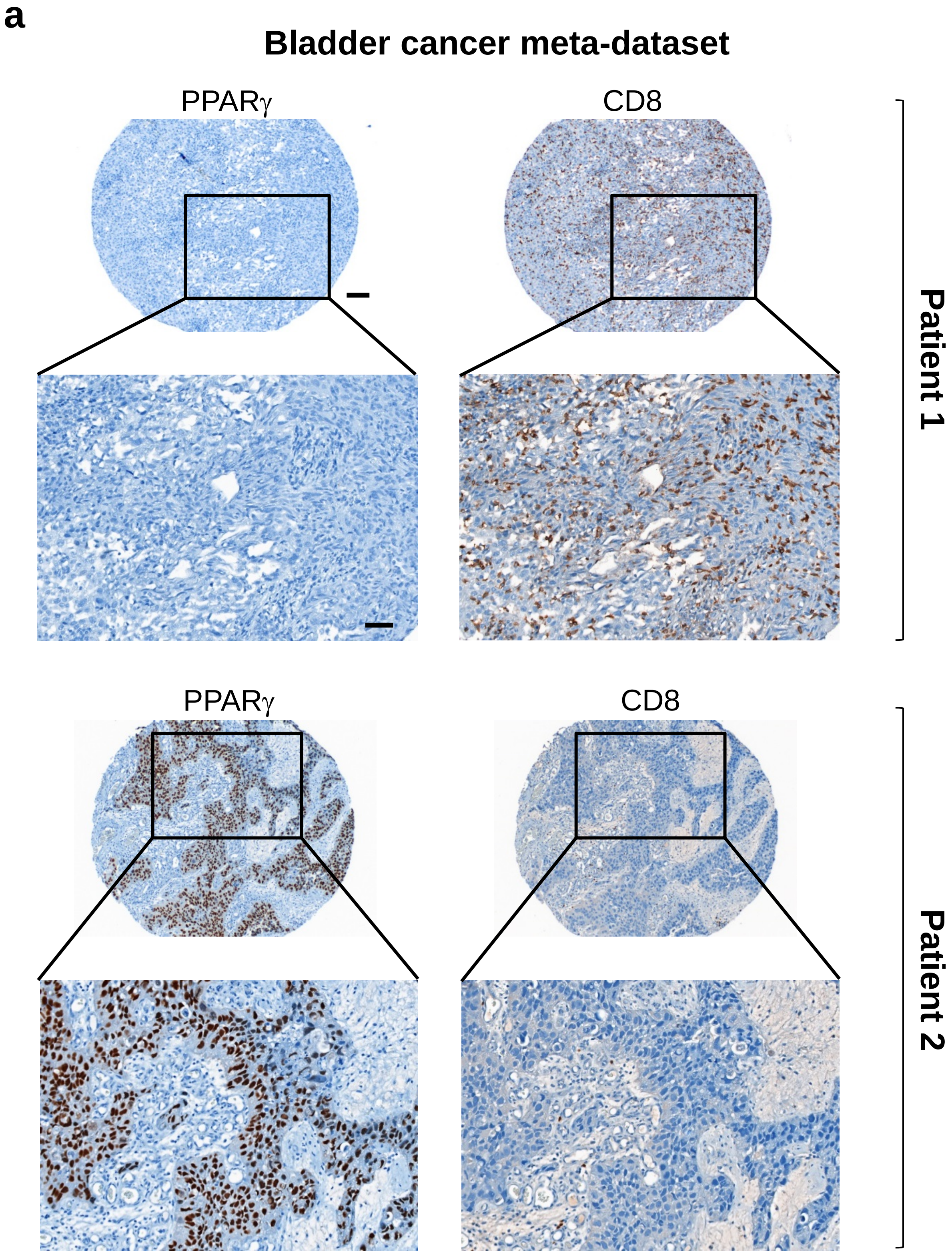

a
Bladder cancer meta-dataset
PPARγ
CD8
Patient 1
PPARγ
CD8
Patient 2

b

Bladder cancer meta-dataset

CD8

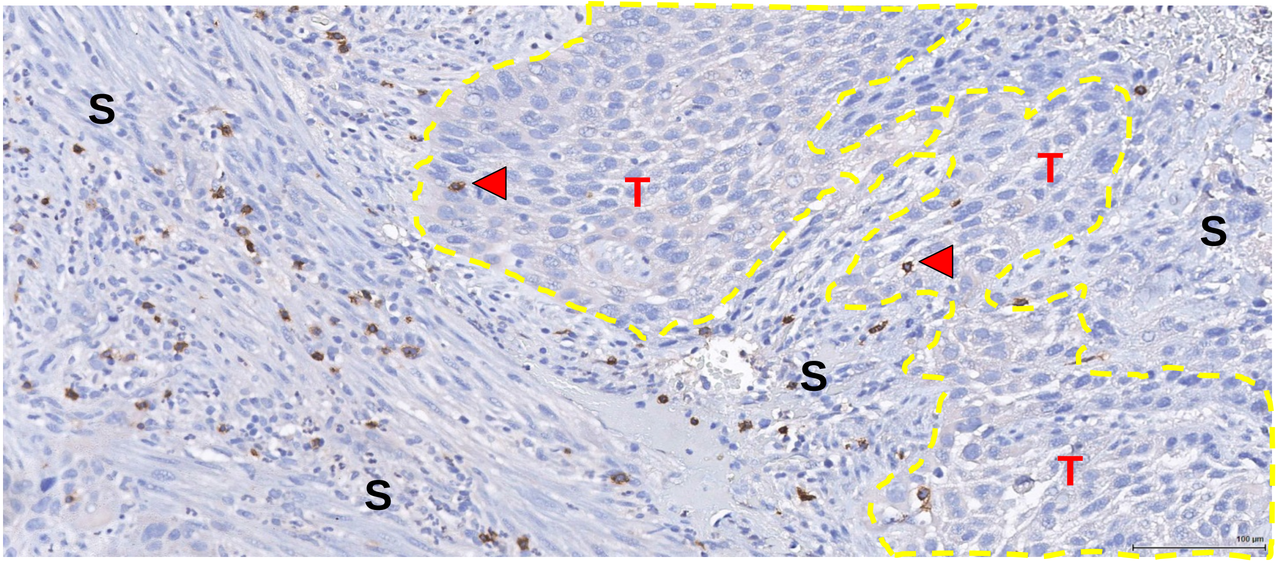

PPARγ

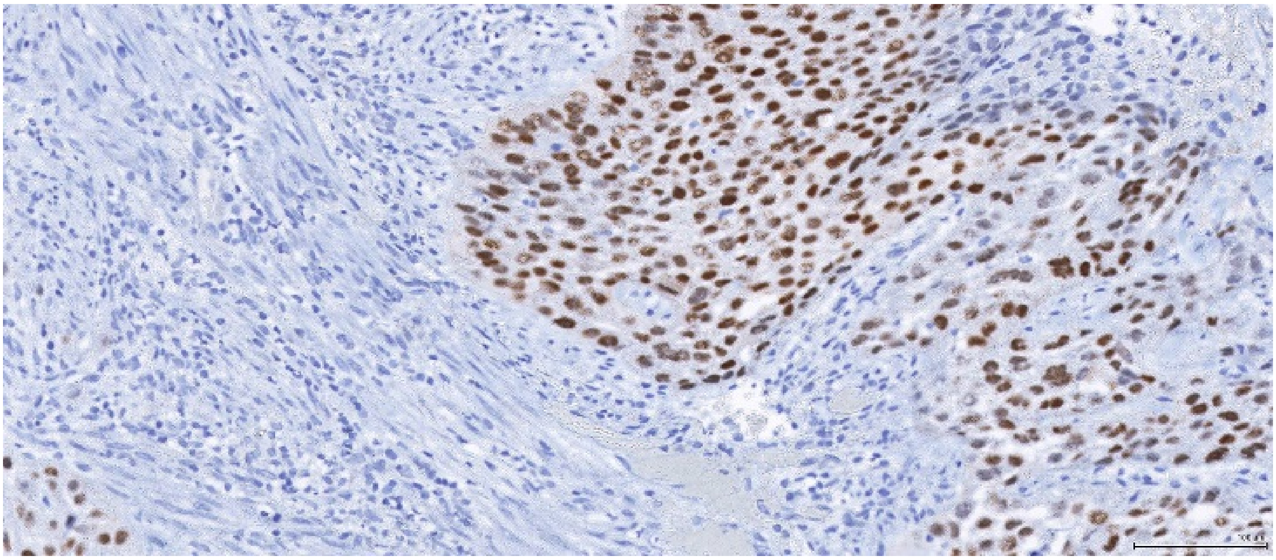

Supplementary Figure 20. Bladder cancer meta-dataset bladder tumor cohort confirms that CD8+ T cell infiltration is negatively correlated with PPARγ expression. (**a**) Representative IHC images of PPARγ and CD8 staining in human bladder tumor samples, back-to-back serial sections from the bladder caner meta-dataset cohort. The upper panel shows a bladder cancer case with negative PPARγ expression where CD8+ T cell infiltration is abundant. The lower panel shows absent or very few CD8+ T cell infiltration in PPARγ highly expressed bladder cancer case. The scale bar represents 100 μm (low magnification image) and 50 μm (high magnification image), respectively. (**b**) The upper panel shows localization of CD8+ T cells within the tumor. CD8+ T cells are abundant in the stroma (S) where PPARγ expression is low and scarce in the tumor compartment (T) where PPARγ expression is high. The scale bar represents 100 μm.

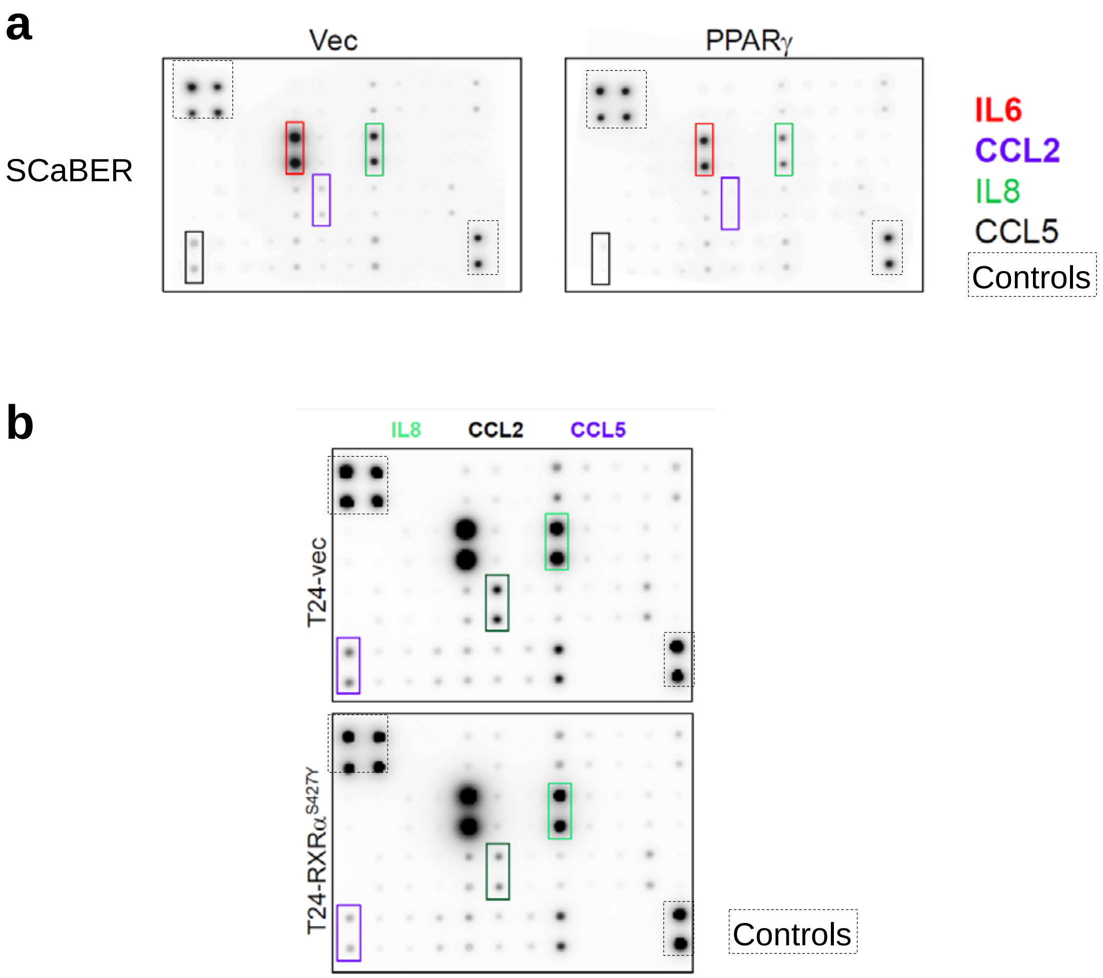

Supplementary Figure 21. Chemokine array analysis of conditioned media collected from human bladder cancer cell lines engineered to overexpress PPARγ, RXRα^{WT} or RXRα^{S427Y}. (**a**) SCaBER cell lines engineered with vector control or PPARγ overexpression. (**b**) T24 cell lines engineered with WT or S427F RXRα. Dotted boxes represent controls. Cytokines/chemokines showing significant changes in secretion are outlined.

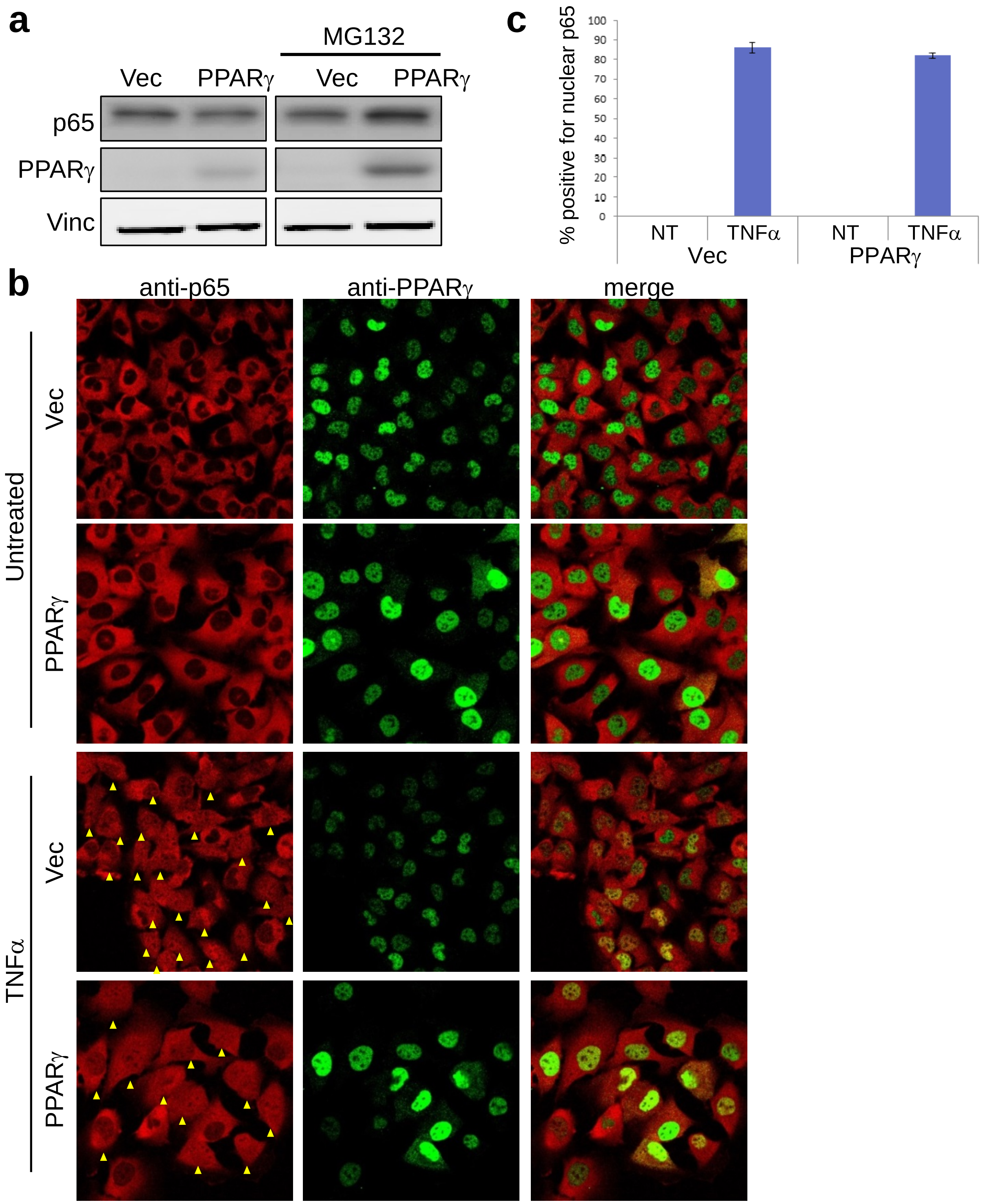

Supplementary Figure 22. Regulation of the NFκB subunit p65 protein by overexpression of PPARγ. (**a**) Western blot analysis of p65 and PPARγ in T24 cells engineered with vector control (Vec) or PPARγ overexpression in the presence or absence of the proteasome inhibitor MG132. No clear difference was observed. (**b**) A confocal microscopy analysis of p65 translocation to the nucleus from the cytoplasm furthermore showed that PPARγ had no substantial effect on NFκB nuclear translocation under untreated conditions or after stimulation by TNFα. Yellow arrows indicate the cells with p65 nuclear staining. (**c**) Bar graph shows the quantification of percentage of Vec or PPARγ overexpressing cells positive for nuclear p65 with or without (NT) TNFα treatment. 100-150 cells were counted and nucleus was define using DAPI staining. Negative cells were identified when p65 staining in the nuclear was absent. Cells were considered positive when the nuclear staining for p65 was present, in the majority of positive cells the nuclear staining were equal or greater than cytoplasm staining.

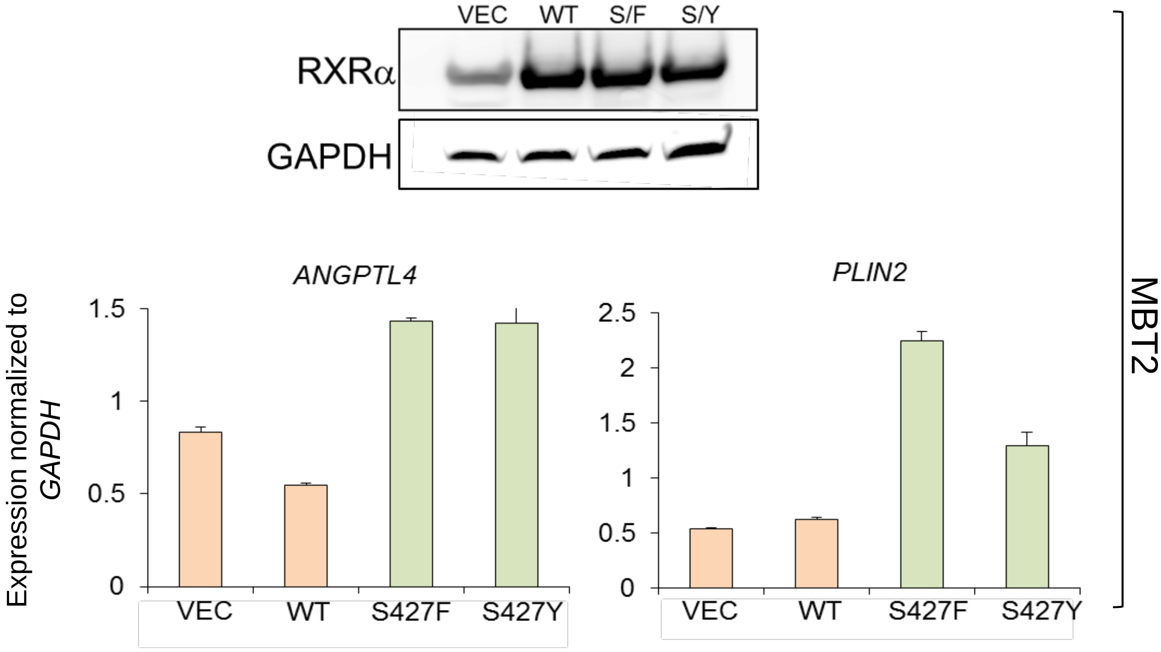

Supplementary Figure 23. Mouse bladder cancer cell line MBT2 engineered for syngeneic model studies *in vivo*. *Upper*, Western blot for RXRα and GAPDH in MBT2 lines engineered to overexpress $RXR\alpha^{WT}$ (WT), $RXR\alpha^{S427F}$ (S/F) and $RXR\alpha^{S427Y}$ (S/Y). *Lower*, RT-qPCR analysis of *ANGPTL4* and *PLIN2* in the vector control (VEC, beige bar), $RXR\alpha^{WT}$ (WT, beige bar), $RXR\alpha^{S427F}$ (S427F, green bar) and $RXR\alpha^{S427Y}$ (S427Y, green bar) MBT2 lines.

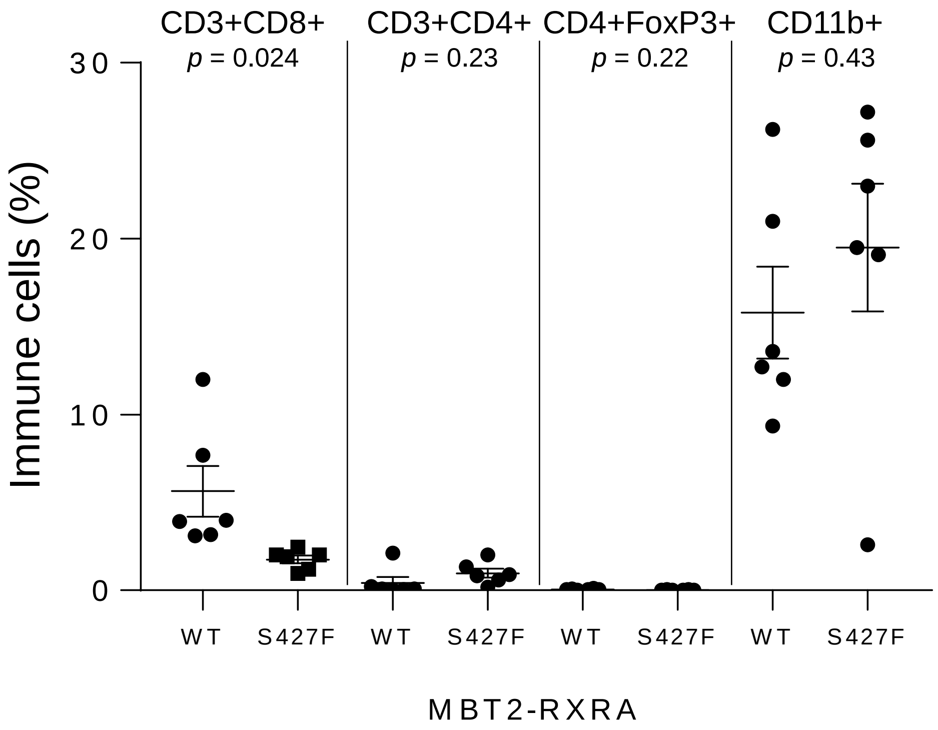

Supplementary Figure 24. FACS based quantitation of infiltrating immune cells into subcutaneously implanted MBT2 tumors overexpressing RXRA-WT (*n* = 6) or RXRA-S427F (*n* = 6). CD3+CD8+, CD3+CD4+, CD4+FOXP3+ and CD11b+ are used as markers to represent CD8+ CTL, CD4+ T helper, Treg and monocyte/MDSC, respectively. Data presented as percent of total tumor-derived cells following dissociation.

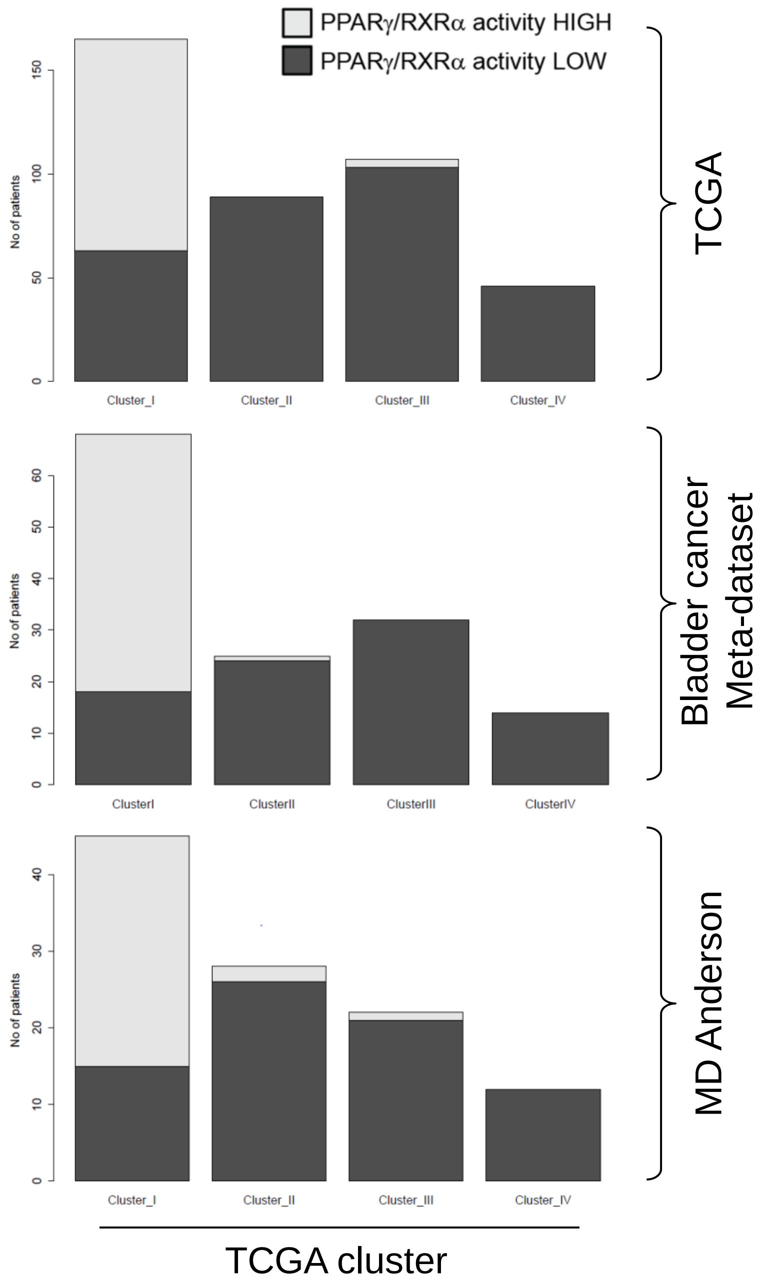

Supplementary Figure 25. PPARγ/RXRα activity in TCGA clusters of MIBCs that are associated with response to anti-PD-L1 treatment in clinic. The PPARγ activity, determined by GLMnet74 was compared between the TCGA clusters. These bar diagrams indicate the PPARγ activity in the TCGA Clusters in 3 different cohorts (TCGA, bladder cancer meta-dataset, and MD Anderson dataset). Tumors assigned to Cluster I showed the highest PPARγ activity in all 3 datasets.

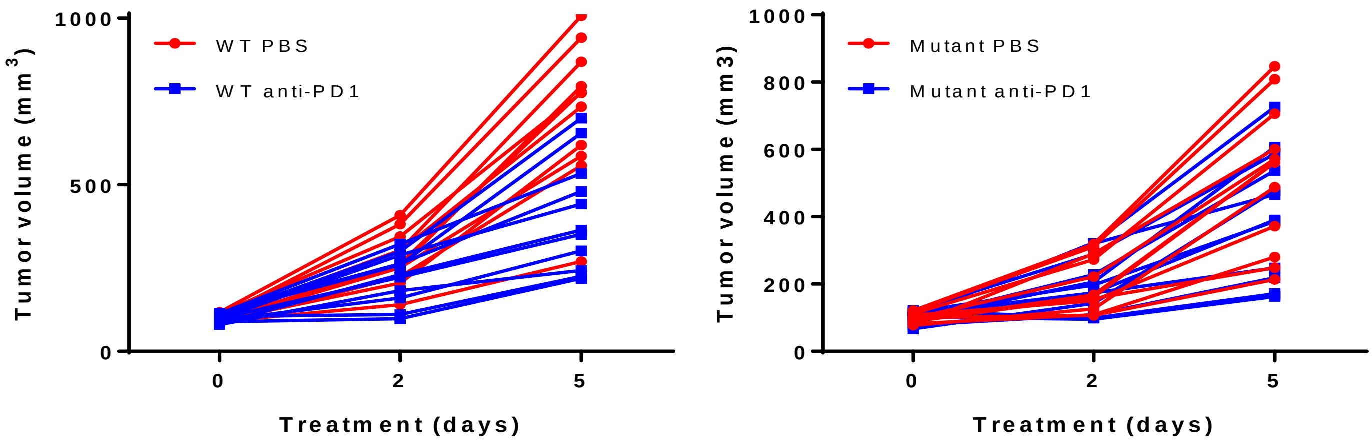

Supplementary Figure 26. RXRα^{S427F} confers partial resistance to anti-PD1 treatment in syngeneic MBT2 models. *Left*, individual MBT2 RXRA-WT tumor volumes in response to PBS (red, n = 12) or anti-PD1 (blue, n = 12). $P < 0.01$ at day 5 of average tumor volume, one-way analysis of variance (ANOVA) followed by the Tukey post-hoc test. *Right*, individual MBT2 RXRA-S427F tumor volumes in response to PBS (red, n = 12) or anti-PD1 (blue, n = 12). $P > 0.05$ at day 5 of average tumor volume, one-way analysis of variance (ANOVA) following by the Tukey post-hoc test.

Fig. 3b

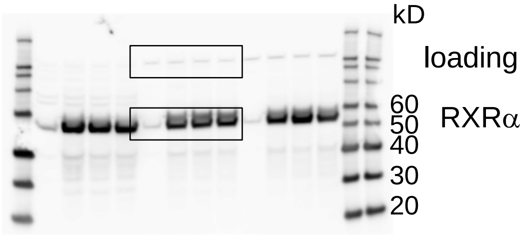

Fig. 3c

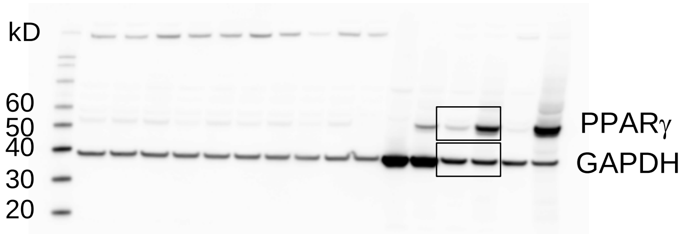

Fig. 3d

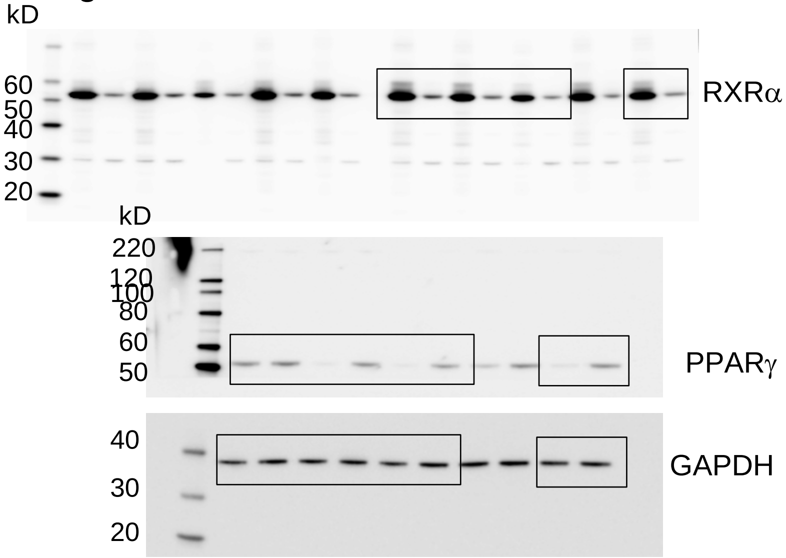

Fig. 5f

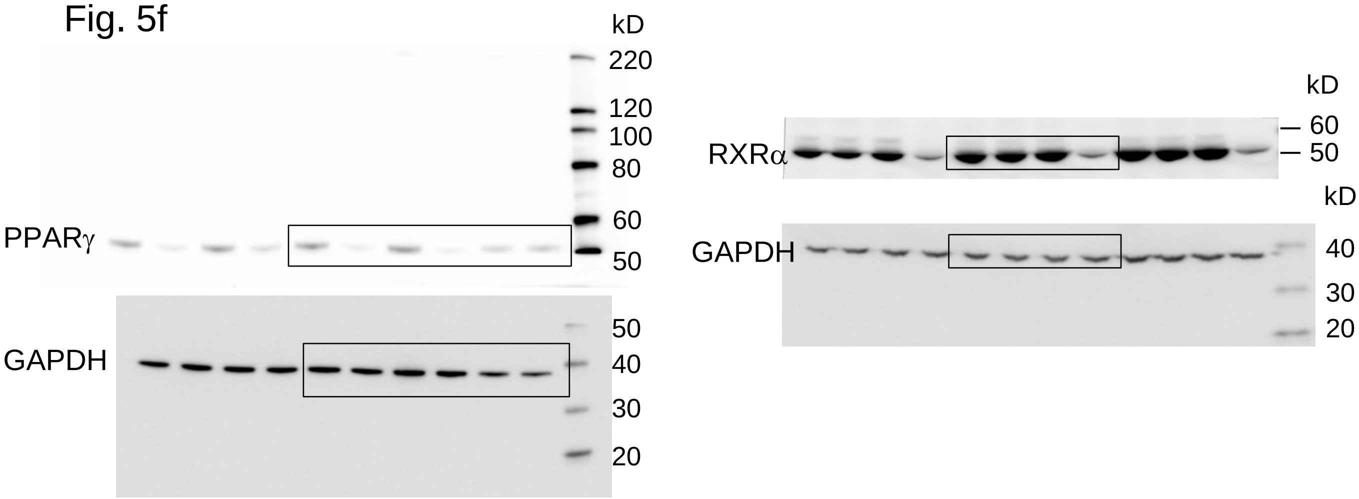

Supplementary Figure 27. Original Western blots.

Supplementary Table 1. Crystallographic Data and Refinement.

Space group	P212121
Unit cell	α = β = γ = 90°; a = 53.9, b = 66.5, c = 165.9 Å
Mosaicity	0.22-0.37
Wavelength	0.9786 Å
Resolution range	45.2-1.98 Å
Unique reflections	42545 (4187)
Multiplicity	7.2 (6.5)
Completeness (%)	100.0 (99.9)
I/σ(I)	21.6 (2.2)
Rmerge[a] (%)	8.9 (86.8)
Rwork[b]/Rfree[c] (%)	19.2/24.6
No residues	513
No waters	231
Av B factor	
Protein	45.3
Ligand	49.8
Water	46.2
Ramachandran (%)	
Most favored	91.2
Allowed	8.4
Generously allowed	0.0
Disallowed	0.4

[a]$R_{merge} = \Sigma|I - \langle I \rangle|/\Sigma I$, where I is the integrated intensity of a given reflection and ⟨I⟩ is the average intensity of multiple observations of symmetry-related reflections.
[b]$R_{work} = \Sigma|F_o - F_c|/\Sigma F_o$), where F_o and F_c are observed and calculated structure factors. [c]R_{free} was calculated from a 5% subset of reflections that were excluded from the refinement.
Parentheses ()'s indicate highest resolution shell.

Supplementary Table 2. List of human tissue samples (Eisai cohort) used for IHC analysis.

Provider ID	Gender	Age	Ethnicity	Clinical Diagnosis	Stage	TNM Stage
10163 T2	M	58	caucasian	bladder cancer	IV	T2aN2M0
10167 T2	M	64	caucasian	bladder cancer	IV	T3bN2M0
10171 T2	F	70	caucasian	bladder cancer	III	T3bN0M0
10172 T2(2)	M	69	caucasian	bladder cancer	IV	T2aN1M0
10174 T2	M	66	caucasian	bladder cancer	IV	T4aN2M0
10175 T2	F	69	caucasian	bladder cancer	III	T3bN0M0
10182 T2	M	57	caucasian	bladder cancer	II	T2bN0M0
10190 T2	M	43	caucasian	bladder cancer	IV	T3bN1M0
10192 T2	M	55	caucasian	bladder cancer	III	T3bN0M0
10198 T2	M	68	caucasian	bladder cancer	III	T4aN0M0
10199 T2	F	66	caucasian	bladder cancer	III	T3aN0M0
10207 T2	M	68	caucasian	bladder cancer	IV	T3aN1M0
10239 T2(2)	M	47	caucasian	bladder cancer	IV	T3aN2M0
10300 T2	M	72	caucasian	bladder cancer	III	T3bN0M0
10376 T2(1)	M	71	caucasian	bladder cancer	IV	T4bN0M0
10388 T2(2)	M	63	caucasian	bladder cancer	III	T4aN0M0
10420 T2(2)	M	52	caucasian	bladder cancer	II	T2bN0M0
10432 T2(2)	F	51	caucasian	bladder cancer	I	T1N0M0
10456 T2(1)	M	58	caucasian	bladder cancer	III	T4aN0M0
10461 T2(1)	M	44	caucasian	bladder cancer	I	T1N0M0
10465 T2(1)	M	75	caucasian	bladder cancer	II	T2bN0M0
10228 T2	M	71	caucasian	bladder cancer	IV	T4bN2M0
10230 T2	M	50	caucasian	bladder cancer	IV	T4aN2M0
10236 T2(2)	M	71	caucasian	bladder cancer	III	T4aN0M0
10237 T2(1)	M	67	caucasian	bladder cancer	III	T4aN0M0

Supplementary Table 3. Summary of qualitative observations made following PPARγ and CD8 IHC staining of the Eisai cohort of human bladder tumors. Staining intensity of PPARγ and localization of CD8+ T cells were noted for each patient sample (unique sample IDs).

Sample ID (Eisai cohort)	PPARγ staining	CD8 staining
10163 T2	Moderate Positive (3)	All in stroma, few tumor
10167 T2	Negative (1)	Both tumor and stroma
10171T2	Negative (1)	Both tumor and stroma
10172T2	Moderate Positive (3)	All in stroma, none in tumor
10174T2	Strong Positive (4)	All in stroma, none in tumor
10175T2	Negative (1)	Both tumor and stroma
10182T2	Weak Positive (2)	Both tumor and stroma
10190T2	Negative (1)	Both tumor and stroma
10192T2	Moderate Positive (3)	All in stroma, none in tumor
10198T2	Moderate Positive (3)	All in stroma, few in tumor
10465T2	Untested	All in stroma, few tumor
10207T2	Strong Positive (4)	All in stroma, none in tumor
10239T2	Negative (1)	Both tumor and stroma
10300T2	Moderate Positive (3)	All in stroma, few tumor
10376T2	Negative (1)	Both tumor and stroma
10388T2	Negative (1)	Both tumor and stroma
10420T2	Negative (1)	All in stroma, none in tumor
10432T2	Weak Positive (2)	All in stroma, none in tumor
10456T2	Very Weak Positive (1)	Both tumor and stroma
10461T2	Strong Positive (4)	All in stroma, none in tumor
10228T2	Negative (1)	None in tumor and stroma
10230T2	Very Weak Positive (1)	Both tumor and stroma
10236T2	Negative (1)	Both tumor and stroma
10237T2	Strong Positive (4)	All in stroma, few tumor

Supplementary Table 4. List of human tissue samples (Bladder cancer meta-dataset cohort) used for IHC analysis.

	Bern (n=65)	Vancouver (n=58)	Southhampton (n=24)	Total (n=146)
Age (median, range) at surgery (years)	63 (35-78)	62 (39-78)	66 (35-81)	63 (35-81)
Gender (female/male)	20/45	16/42	7/17	43/102
**Cystectomy and lymphadenectomy data**				
Tumor stage (n)				
ypT0/1 (%)	24 (37)	24 (41)	9 (38)	57 (39)
ypT2 (%)	12 (18)	13 (23)	6 (24)	31 (21)
ypT3/4 (%)	29 (45)	21 (36)	9 (38)	59 (40)
Lymph node stage (n)				
ypN0 (%)	37 (57)	51 (88)	19 (79)	107 (73)
ypN+ (%)	28 (43)	7 (12)	5 (21)	40 (27)

Supplementary Table 5. Sequences of primers and reagent information.

shRNAs	Sequences
PPARG_shRNA#4 Forward	5'CCGGCAAGTAACTCTCCTCAAATATCTCGAGATATTTGAGGAGAGTTACTTGTTTTTG-3’
PPARG_shRNA#4 Reverse	5'-AATTCAAAAACAAGTAACTCTCCTCAAATATCTCGAGATATTTGAGGAGAGTTACTTG-3'
PPARG_shRNA#5 Forward	5'-CCGGCTGGCCTCCTTGATGAATAAACTCGAGTTTATTCATCAAGGAGGCCAGTTTTTG-3’
PPARG_shRNA#5 Reverse	5'-AATTCAAAAACTGGCCTCCTTGATGAATAAACTCGAGTTTATTCATCAAGGAGGCCAG-3'
PPARG_shRNA#9 Forward	5'-CCGGATCTGACACCTAAGAAATTTACTCGAGTAAATTTCTTAGGTGTCAGATTTTTTG-3'
PPARG_shRNA#9 Reverse	5'-AATTCAAAAAATCTGACACCTAAGAAATTTACTCGAGTAAATTTCTTAGGTGTCAGAT-3’
RXRA-shRNA Forward	5’-CCGGGTGTTGTCACCCTCCTTATTTCTCGAGAAATAAGGAGGGTGACAACACTTTTTG-3’
RXRA-shRNA Reverse	5’-AATTCAAAAAGTGTTGTCACCCTCCTTATTTCTCGAGAAATAAGGAGGGTGACAACAC-3’

Human Taqman Probes	Cat #
ANGPTL4	Hs01101127_m1
PLIN2	Hs00605340_m1
PSCA	Hs04177224_g1
PDK4	Hs01037712_m1
ACOX1	Hs01074241_m1
IL6	Hs00985639_m1
IL8	Hs00174103_m1
IL18	Hs01038788_m1
CCL2	Hs00234140_m1
CCL5	Hs00982282_m1
TNF	Hs01113624_g1

Mouse Taqman Probes	Cat #
Angptl4	Mm00480431_m1
Plin2	Mm00475794_m1

	For RXRα cDNA	For PPARγ cDNA	For shRNA
	G418 (mg/ml)	Balsticidin (mg/ml)	Puromycin (mg/ml)
KU-19-19	400		
MBT2	400	25	
SCaBER	1500	10	
T24	800	25	
SV-HUC-1	400		0.5
HT-1197			1.5
5637			0.5
